# Lifestyle factors and clinical severity of Parkinson’s disease

**DOI:** 10.1038/s41598-023-31531-w

**Published:** 2023-06-12

**Authors:** Carolin Gabbert, Inke R. König, Theresa Lüth, Meike Kasten, Anne Grünewald, Christine Klein, Joanne Trinh

**Affiliations:** 1 Institute of Neurogenetics, University of Lübeck, Ratzeburger Allee 160, 23538 Lübeck, Germany; 2 Institute of Medical Biometry and Statistics, University of Lübeck, Lübeck, Germany; 3 Department of Psychiatry and Psychotherapy, University of Lübeck, Lübeck, Germany; 4 Luxembourg Centre for Systems Biomedicine, University of Luxembourg, Esch-Sur-Alzette, Luxembourg

**Keywords:** Environmental impact, Neurodegenerative diseases, Parkinson's disease, Risk factors, Signs and symptoms

## Abstract

Genetic factors, environmental factors, and gene–environment interactions have been found to modify PD risk, age at onset (AAO), and disease progression. The objective of this study was to explore the association of coffee drinking, aspirin intake, and smoking, with motor and non-motor symptoms in a cohort of 35,959 American patients with PD from the Fox Insight Study using generalized linear models. Coffee drinkers had fewer problems swallowing but dosage and duration of coffee intake were not associated with motor or non-motor symptoms. Aspirin intake correlated with more tremor (p = 0.0026), problems getting up (p = 0.0185), light-headedness (p = 0.0043), and problems remembering (p = 1 × 10^–5^). Smoking was directly associated with symptoms: smokers had more problems with drooling (p = 0.0106), swallowing (p = 0.0002), and freezing (p < 1 × 10^–5^). Additionally, smokers had more possibly mood-related symptoms: unexplained pains (p < 1 × 10^–5^), problems remembering (p = 0.0001), and feeling sad (p < 1 × 10^–5^). Confirmatory and longitudinal studies are warranted to investigate the clinical correlation over time.

## Introduction

An interplay of genetic and environmental factors is known to influence age at onset (AAO) in Parkinson’s disease (PD)^1^, the risk for PD, as well as PD progression, and the severity of symptoms^2–4^. However, studies that focus on the severity of motor and non-motor symptoms are sparse. Most of them investigate motor and non-motor symptoms by separating participants based on their current coffee drinking or smoking behavior without differentiating between use before or after disease onset^5–9^. Additionally, cohort sizes range between 100 and 300 patients with PD^5–9^, illustrating the importance to investigate the interaction between lifestyle factors and motor symptoms in larger cohorts for better statistical power. There are indications of a protective effect of coffee on motor and non-motor symptoms. An inverse association has been found between the consumption of coffee and the severity of non-motor symptoms related to mood and cognition^6^. Additionally, it was reported that coffee drinkers had lower tremor scores compared to non-coffee drinkers, also with a dose-dependent relationship between coffee consumption and tremor severity, but this was only significant in male patients with de novo PD^7^. In contrast to the findings for coffee, no differences in motor or non-motor symptom severity were reported between smokers and non-smokers. There was no significant difference in the duration from motor symptom onset to reaching Hoehn and Yahr stage 3 between smokers and non-smokers^8^. Additionally, no significant differences were reported in change of disease severity, symptoms of depression, and cognitive impairment between smokers and non-smokers^5^. In accordance with this, no associations between the Non-Motor Symptoms Questionnaire (NMSQ) total score and smoking status were found. However, following a multinomial logistic regression stepwise model using never smoking as a reference, several non-motor symptoms showed associations with current or former smoking, e.g. lower Unified Parkinson’s Disease Rating Scale (UPDRS) part III total scores were associated with smoking^9^.

Then again, other studies aim to investigate the immediate effect of coffee or smoking on PD symptoms by specifically administering caffeine or nicotine, which are the suspected agents responsible for the effect of coffee drinking and smoking, respectively. However, studies investigating the specific treatment with caffeine and nicotine after disease onset did not indicate any association with motor symptoms, as motor scores did not significantly differ between treated patients and untreated patients^10,11^. In contrast to coffee and smoking, a possible immediate association of aspirin with motor symptoms or motor symptom severity in PD has yet to be investigated.

Tobacco use, coffee consumption, and aspirin intake have already been found to be associated with AAO in the Fox Insight American PD cohort^1^. However, how these specific lifestyle factors affect PD motor and non-motor symptoms remains unclear, especially when strictly exploring the intake before disease manifestation. Herein, we performed a cross-sectional exploration of the association of the consumption of coffee, smoking, and the use of aspirin on motor and non-motor symptoms in patients with PD.

## Methods

### Demographics and participant examination

Our study sample consists of 35,959 American patients with PD (Supplementary Table S1) from the Fox Insight study (Supplementary Text and Supplementary Figure S1). The Fox Insight study is an ongoing online, longitudinal health study of people with and without PD^12^. The dataset is generated through routine longitudinal assessments, one-time health and disease questionnaires about symptoms, daily activities, and other factors, and, in a subgroup of people with PD, genetic data collection. Fox Insight participants were 18 years of age or older and provided informed consent. In the process of registration, participants were divided into two groups, PD patients and controls, the latter were asked about new diagnoses every three months. PD patients responded to health, non-motor assessments, motor assessments, quality of life, and lifestyle questionnaires. All analyses were performed in accordance with relevant guidelines and regulations. Data collection was performed via an online data platform. We excluded patients with PD with an AAO lower than 3 years as well as with an age at examination (AAE) lower than 18 years. Most of the patients were of white/Caucasian ethnicity (89.9%) (Supplementary Table S1). PD patients had a mean AAE of 65.7 ± 10.2 SD years (range 18.1–119.0 years) and a mean AAO of 60.4 ± 11.0 SD years (range 5.1–115.4 years). The mean disease duration until examination at Fox Insight was 5.3 ± 5.6 SD years (range 0–64.3 years) and the mean disease duration until their current age was 6.5 ± 5.7 SD years (range 0–64.3 years); 40.4% of PD patients were female.

Clinical variables were downloaded from Fox Insight questionnaires: “Your Movement Experiences”, “Your Non-movement Experiences”, “Your Current Health”, and “Your Mood”. Patients report tremor, speech impairment, excess of saliva and drooling, problems chewing and swallowing, problems walking and balance, freezing, and problems getting up for “Your Movement Experiences”. Constipation, unexplained pains, problems remembering, feeling sad, feeling anxious, changed interest in sex, and lightheadedness were reported as “Your Non-movement Experiences”. More specific variables on mood were assessed with “Your Current Health” and “Your Mood” questionnaires. These correspond to participant questionnaires MDS-UPDRS II, NMSQ, and Geriatric Depression Scale (GDS). The PD Risk Factor Questionnaires (PD-RFQ-Us) were used for lifestyle and environmental factors. Each questionnaire and specific variables within are described in detail in the Supplementary Text and Supplementary Table S2.

### Lifestyle factors

The intake of coffee, aspirin, and tobacco was estimated from Environmental Exposure Questionnaires and the definitions of coffee drinkers, aspirin users, and smokers were previously described for this cohort^1^. Patients were classified as coffee drinkers if they drank caffeinated coffee at least once per week over a minimum period of six months. Patients were classified as aspirin users if they took at least two pills of aspirin per week for at least six months. Lastly, patients were classified as smokers/tobacco users, if they smoked more than 100 cigarettes in their lifetime or if they smoked at least one cigarette per day over a period of at least six months, or if they used smokeless tobacco at least once per day for more than six months.

To assess dosage and long-term effects, duration of caffeine consumption, aspirin intake, and smoking were estimated according to the age the patients started using either substance subtracted from the age at termination or from their AAO if the patients terminated the consumption after their AAO. Periods in between, where the patients stopped regularly consuming, were not included in the duration. Coffee drinking dosage was defined as cups of coffee per week within coffee drinking duration, excluding all values higher than 100 cups per week from the analysis. Aspirin dosage was defined as aspirin pills per week the patients took within aspirin intake duration time. Smoking dosage was estimated as cigarettes smoked per day during time smoking, excluding implausible values (> 100 cigarettes per day). For dosage analyses, the number of cups of coffee for non-drinkers, pills per week for aspirin non-users, and cigarettes for non-smokers was set to zero.

### Statistical analysis

Generalized linear regression models were used to estimate the relationship between environmental factors, age, gender, disease duration, and motor/non-motor symptoms (R studio) (details of each model are in the Supplementary Text). Reported p values remain descriptive because they are not corrected for multiple testing and results are exploratory. Patients with missing data on the use of environmental and lifestyle factors or motor and non-motor symptoms were not included in the analyses. Numbers of patients included in each regression model are reported in the tables.

Multiple regression models were estimated to predict the respective symptoms to assess the relationship between environmental factors and motor/non-motor symptoms. To explore potential confounders, we adjusted for covariates by including AAE, gender, disease duration, and potential comorbidities. Environmental factors were separately handled in three different ways: (1) binary (yes–no indication), (2) dosage as a continuous variable, and (3) duration as a continuous variable. Patients indicating no for lifestyle factor had values set to zero. Motor symptoms were dichotomized (yes: scores > 1 or no: score = 1) because data were not normally distributed (details in Supplementary Text).

### Ethics approval

All participants provided informed consent using the Fox Insight website. The Fox Insight study has been approved by the New England Institutional Review board (IRB) (IRB: 120160179; Legacy IRB#: 14–236, Sponsor Protocol Number: 1, Study Title: Fox Insight). Approval was obtained from the Ethics Committee of the University of Lübeck. We confirm that all analyses were performed in accordance with relevant guidelines and regulations.

### Consent to participate

Informed consent was obtained from all individual participants included in the study.

## Results

### Coffee

We first explored the association of coffee drinking before AAO with self-reported motor and non-motor features (Supplementary Table S2). When coffee drinking was used as a binary yes–no indication in a regression model with covariates AAE, gender, and disease duration (Table 1, Supplementary Table S3) coffee drinkers had fewer problems with chewing and swallowing (p = 0.0497, β = − 0.1435, SE = 0.0731). When cups of coffee per week or coffee drinking duration were used as continuous variables, we found no association with any of the motor symptoms (Table 1).

**Table 1:** Motor symptoms associated with environmental factors in regression models.

	Coffee			Aspirin			Smoking		
	Yes/no	Dosage	Duration	Yes/no	Dosage	Duration	Yes/no	Dosage	Duration
Tremor									
n	4889	3848	1967	2866	2730	547	5269	4201	876
p value	0.5797	0.0559	0.3860	**0.0026**	**0.0138**	0.5498	0.1366	0.3171	0.5973
β	− 0.0473	0.0084	− 0.0048	0.3174	0.0287	0.0064	0.1038	0.0038	− 0.0035
Speech									
n	4892	3849	1967	2868	2732	547	5272	4203	876
p value	0.1615	0.7086	0.2585	0.5007	0.0880	0.4461	0.1330	**0.0062**	**0.0454**
β	− 0.1021	− 0.0013	0.0050	− 0.0582	0.0152	− 0.0062	0.0893	0.0089	0.0119
Saliva and drooling									
n	4892	3849	1967	2868	2732	547	5272	4203	876
p value	0.0823	0.9754	0.9591	0.2339	0.3290	0.5824	**0.0106**	**0.0022**	0.0976
β	− 0.1232	0.0001	− 0.0002	0.0997	0.0080	0.0044	0.1484	0.0096	0.0093
Chewing and swallowing									
n	4892	3849	1967	2868	2732	547	5272	4203	876
p value	**0.0497**	0.6961	0.4785	**0.0358**	**0.0182**	0.9015	**0.0002**	** < 1 × 10**^**–5**^	0.0589
β	− 0.1435	− 0.0014	0.0033	0.1837	0.0201	− 0.0010	0.2243	0.0174	0.0107
Walking and balance									
n	4889	3848	1967	2866	2730	547	5269	4201	876
p value	0.1156	0.8287	0.6311	0.1056	**0.0106**	0.8477	0.2660	**0.0038**	**4 × 10**^**–5**^
β	− 0.1223	− 0.0008	0.0022	0.1478	0.0253	− 0.0017	0.0698	0.0101	0.0268
Freezing									
n	4889	3848	1967	2866	2730	547	5269	4201	876
p value	0.2364	0.7292	0.9616	0.2979	0.1481	0.9126	**0.0212**	**0.0052**	** < 1 × 10**^**–5**^
β	− 0.0935	0.0013	0.0002	0.0996	0.0133	0.0010	0.1490	0.0094	0.0277
Getting up									
n	4889	3848	1967	2866	2730	547	5269	4201	876
p value	0.4886	0.5482	0.7142	**0.0185**	**0.0182**	0.3039	0.0855	**0.0061**	** < 1 × 10**^**–5**^
β	− 0.0540	− 0.0022	0.0017	0.2170	0.0231	− 0.0088	0.1099	0.0098	0.0366

p value (exploratory): multivariate regression to predict the respective motor symptoms adjusted for covariates by including AAE, gender, and disease duration (time between AAO and current age) in the model.

Significant values are in [bold].

Furthermore, coffee did not show an association with the available non-motor symptoms (p > 0.05) (Table 2, Supplementary Table S4). When cups of coffee per week were used as a continuous variable, coffee drinking dosage showed a direct correlation with unexplained pains (p = 0.0168, β = 0.0083, SE = 0.0035) (Table 2, Supplementary Table S5). Here, the higher the dosage of coffee, the more unexplained pains were experienced.

**Table 2:** Non-motor symptoms associated with environmental factors in regression models.

	Coffee			Aspirin			Smoking		
	Yes/no	Dosage	Duration	Yes/no	Dosage	Duration	Yes/no	Dosage	Duration
Constipation									
n	4917	3868	1977	2881	2743	548	5297	4221	880
p value	0.0970	0.9669	0.4438	**0.0124**	**0.0037**	0.6783	0.1994	0.2026	0.6233
β	− 0.1164	− 0.0001	0.0033	0.2077	0.0251	0.0033	0.0735	0.0039	0.0027
Unexplained pains									
n	4917	3868	1977	2881	2743	548	5296	4221	880
p value	0.1623	**0.0168**	0.0883	**0.0227**	**0.0320**	0.8718	** < 1 × 10**^**–5**^	**0.0003**	**0.0134**
β	0.1017	0.0083	0.0080	0.1961	0.0178	− 0.0013	0.2732	0.0114	0.0140
Problems remembering									
n	4917	3868	1977	2881	2743	548	5296	4221	880
p value	0.9859	0.7212	0.5348	**1 × 10**^**–5**^	**0.0005**	0.5864	**0.0001**	**6 × 10**^**–5**^	**0.0017**
β	0.0012	0.0012	− 0.0027	0.3662	0.0295	0.0043	0.2176	0.0123	0.0176
Feeling sad									
n	4914	3865	1976	2880	2742	548	5293	4218	880
p value	0.2517	0.0627	0.5381	0.0665	**0.0344**	0.4675	** < 1 × 10**^**–5**^	** < 1 × 10**^**–5**^	**0.0470**
β	0.0804	0.0063	0.0027	0.1537	0.0177	0.0058	0.3279	0.0152	0.0112
Anxiety									
n	4914	3865	1976	2880	2742	548	5293	4218	880
p value	0.2199	0.1432	0.5958	0.2999	0.1574	0.8303	** < 1 × 10**^**–5**^	** < 1 × 10**^**–5**^	0.3910
β	0.0902	0.0052	0.0025	0.0920	0.0118	− 0.0018	0.3007	0.0161	0.0049
Changed interest in sex									
n	4914	3865	1976	2880	2742	548	5293	4218	880
p value	0.1032	0.0505	0.6957	**0.0221**	**0.0278**	0.7925	**0.0013**	0.2351	**0.0372**
β	0.1227	0.0069	0.0018	0.2023	0.0184	0.0022	0.1959	0.0038	0.0123
Light-headedness									
n	4913	3864	1976	2880	2742	548	5292	4217	880
p value	0.2808	0.0561	0.1935	**0.0043**	**0.0060**	0.5288	**0.0005**	**0.0001**	0.1326
β	0.0758	0.0064	− 0.0056	0.2380	0.0226	0.0050	0.2000	0.0117	0.0083

p value (exploratory): multivariate regression to predict the respective non-motor symptoms adjusted for covariates by including AAE, gender, and disease duration (time between AAO and current age) in the model.

Significant values are in [bold].

### Aspirin

We further explored the association of aspirin intake before AAO with self-reported motor features (Supplementary Table S2). Aspirin intake (binary yes–no indication) showed a direct association with tremor (p = 0.0026, β = 0.3174, SE = 0.1054), chewing and swallowing problems (p = 0.0358, β = 0.1837, SE = 0.0875), and getting up (p = 0.0185, β = 0.2170, SE = 0.0922) (Table 1, Supplementary Table S3), indicating a higher probability to have tremor or problems with swallowing or getting out of a bed or a chair in the group of aspirin users compared to non-users. We further investigated potential comorbidities by including heart diseases, arthritis, back pain, and surgeries with anesthesia in the regression models. The exploratory association of aspirin intake with most motor symptoms was still robust after including the comorbidities (Supplementary Table S6) with the exception of chewing and swallowing: the association diminished when heart diseases (p = 0.1120, β = 0.1425, SE = 0.0897), arthritis (p = 0.0556, β = 0.1684, SE = 0.0880), back pain (p = 0.0591, β = 0.1661, SE = 0.0880), and surgeries with anesthesia (p = 0.0591, β = 0.1661, SE = 0.0880) were included (Supplementary Table S7). In addition, the association between aspirin intake and getting up diminished when heart diseases were included in the model (p = 0.0534, β = 0.1819, SE = 0.0941) (Supplementary Table S7).

When assessing dosage effects, more aspirin taken per week associated with more problems with tremor (p = 0.0138, β = 0.0287, SE = 0.0117), chewing and swallowing (p = 0.0182, β = 0.0201, SE = 0.0085), walking and balance (p = 0.0106, β = 0.0253, SE = 0.0099), and getting up (p = 0.0182, β = 0.0231, SE = 0.0098) (Table 1, Supplementary Table S3). When potential confounding comorbidities were investigated, the association between the aspirin intake dosage and getting up diminished when back pain was included in the model (p = 0.0509, β = 0.0196, SE = 0.0100) (Supplementary Tables S6, S7).

Aspirin intake duration did not show an association with any of the motor symptoms (Table 1).

In addition, we explored the association between aspirin intake status and non-motor symptoms (Table 2, Supplementary Tables S2, S4). Aspirin intake (binary yes–no indication) exhibited a direct association with constipation (p = 0.0124, β = 0.2077, SE = 0.0831), unexplained pains (p = 0.0227, β = 0.1961, SE = 0.0861), problems remembering (p = 1 × 10^–5^, β = 0.3662, SE = 0.0830), changed interest in sex (p = 0.0221, β = 0.2023, SE = 0.0884), and light-headedness (p = 0.0043, β = 0.2380, SE = 0.0833) (Table 2, Supplementary Table S5). Thus, the odds of experiencing constipation, unexplained pains, problems remembering, a changed interest in sex, or feeling light-headed were higher for aspirin users. When more aspirin pills per week were taken, more problems with non-motor symptoms such as constipation (p = 0.0037, β = 0.0251, SE = 0.0086), unexplained pains (p = 0.0320, β = 0.0178, SE = 0.0083), problems remembering (p = 0.0005, β = 0.0295, SE = 0.0085), feeling sad (p = 0.0344, β = 0.0177, SE = 0.0083), changed interest in sex (p = 0.0278, β = 0.0184, SE = 0.0084), and light-headedness (p = 0.0060, β = 0.0226, SE = 0.0082) (Table 2, Supplementary Table S5) were reported. The association between aspirin intake dosage and unexplained pains diminished when arthritis (p = 0.0672, β = 0.0154, SE = 0.0084) and back pain (p = 0.0896, β = 0.0142, SE = 0.0084) were included (Supplementary Tables S8, S9). In addition, the association between aspirin intake dosage and feeling sad diminished when including heart diseases (p = 0.0806, β = 0.0147, SE = 0.0084), arthritis (p = 0.0513, β = 0.0164, SE = 0.0084), and back pain (p = 0.0527, β = 0.0163, SE = 0.0084) (Supplementary Tables S8, S9). All other motor and non-motor symptoms remained associated with aspirin intake. Aspirin intake duration did not show an association with any of the non-motor symptoms (Table 2).

### Smoking

Lastly, we explored the association of smoking before AAO with self-reported motor features (Supplementary Table S2). Smoking directly correlated with excessive saliva and drooling (p = 0.0106, β = 0.1484, SE = 0.0580), chewing and swallowing problems (p = 0.0002, β = 0.2243, SE = 0.0603), and freezing (p = 0.0212, β = 0.1490, SE = 0.0646), when smoking was used as a binary yes–no indication (Table 1, Supplementary Table S3), indicating that smokers had more problems with too much saliva, problems with swallowing, and freezing. In addition, smoking dosage correlated with more problems with speech (p = 0.0062, β = 0.0089, SE = 0.0033), saliva excess and drooling (p = 0.0022, β = 0.0096, SE = 0.0031), chewing and swallowing (p < 1 × 10^–5^, β = 0.0174, SE = 0.0031), walking and balance (p = 0.0038, β = 0.0101, SE = 0.0035), freezing (p = 0.0052, β = 0.0094, SE = 0.0034), and getting up (p = 0.0061, β = 0.0098, SE = 0.0036) (Table 1, Supplementary Table S3). Smoking duration also correlated with more problems with speech (p = 0.0454, β = 0.0119, SE = 0.0059), walking and balance (p = 4 × 10^–5^, β = 0.0268, SE = 0.0065), freezing (p < 1 × 10^–5^, β = 0.0277, SE = 0.0062), and getting up (p < 1 × 10^–5^, β = 0.0366, SE = 0.0070) (Table 1, Supplementary Table S3). Thus, more problems with motor symptoms were observed when more cigarettes were smoked per day and when smoking duration was longer. However, when investigating potential comorbidities, the association between smoking duration and speech diminished when heart diseases (p = 0.0526, β = 0.0115, SE = 0.0059) and lung diseases (p = 0.0591, β = 0.0113, SE = 0.0060) were included in the models, although heart diseases (p = 0.7074, β = 0.0752, SE = 0.2002) and lung diseases (p = 0.5828, β = 0.1105, SE = 0.2011) showed no association with speech either (Supplementary Tables S7, S10).

Most strikingly, smoking was directly associated with non-motor symptoms (Table 2, Supplementary Tables S2, S4). Smokers experienced more unexplained pains (p < 1 × 10^–5^, β = 0.2732, SE = 0.0595), problems remembering (p = 0.0001, β = 0.2176, SE = 0.0570), feeling sad (p < 1 × 10^–5^, β = 0.3279, SE = 0.0579), anxiety (p < 1 × 10^–5^, β = 0.3007, SE = 0.0604), changed interest in sex (p = 0.0013, β = 0.1959, SE = 0.0610), and light-headedness (p = 0.0005, β = 0.2000, SE = 0.0574) (Table 2, Supplementary Table S5). Smoking dosage correlated with more unexplained pains (p = 0.0003, β = 0.0114, SE = 0.0031), problems remembering (p = 6 × 10^–5^, β = 0.0123, SE = 0.0030), feeling sad (p < 1 × 10^–5^, β = 0.0152, SE = 0.0031), anxiety (p < 1 × 10^–5^, β = 0.0161, SE = 0.0031), and light-headedness (p = 0.0001, β = 0.0117, SE = 0.0030) (Table 2, Supplementary Table S5). Smoking duration also correlated with more unexplained pains (p = 0.0134, β = 0.0140, SE = 0.0057), problems remembering (p = 0.0017, β = 0.0176, SE = 0.0056), feeling sad (p = 0.0470, β = 0.0112, SE = 0.0056), and changed interest in sex (p = 0.0372, β = 0.0123, SE = 0.0059) (Table 2, Supplementary Table S5). Thus, more problems with non-motor symptoms were observed with more cigarettes smoked per day and longer smoking duration. However, the association between smoking duration and feeling sad diminished when heart diseases (p = 0.0807, β = 0.0099, SE = 0.0057) and lung diseases (p = 0.1103, β = 0.0091, SE = 0.0057) were included in the model. In addition, the association with a changed interest in sex (p = 0.0574, β = 0.0113, SE = 0.0059) diminished when heart diseases were included, although heart diseases itself showed no association with a changed interest in sex either (p = 0.0795, β = 0.3482, SE = 0.1986) (Supplementary Tables S9, S11).

As the most prominent association for smoking with non-motor symptoms, we additionally explored symptoms specifically related to mood (Supplementary Table S2). More smokers (binary yes–no indication) exhibited depression (p < 1 × 10^–5^, β = 0.3362, SE = 0.0649), anxiety (p = 2 × 10^–5^, β = 0.2748, SE = 0.0636), and other factors associated with depression and mood. These include: dropped many activities and interests, life feels empty, getting bored often, being afraid something bad could happen, feeling helpless often, prefer staying at home, feeling to have more memory problems than other people, feeling pretty worthless, and feeling that situation is hopeless (p < 0.0025, β > 0.1955) (Table 3, Supplementary Tables S12, S13). Likewise, cigarettes smoked per day are associated with more depression (p < 1 × 10^–5^, β = 0.0199, SE = 0.0033), anxiety (p < 1 × 10^–5^, β = 0.0182, SE = 0.0032), as well as factors associated with depression and mood (p < 0.0014, β > 0.0105). Smoking duration also showed the same direction for depression (p = 0.0422, β = 0.0123, SE = 0.0061), and factors associated with depression (p < 0.0039, β > 0.0178) (Table 3, Supplementary Table S13). Thus, all investigated symptoms related to depression and mood were associated with smoking indicating that the higher the smoking dosage and duration, the more likely it was that patients with PD experienced a negative mood. When potential confounding comorbidities were investigated, all symptoms related to mood remained associated with smoking with the exception of depression: the association between smoking duration and depression diminished when heart diseases (p = 0.0544, β = 0.0117, SE = 0.0061) and lung diseases (p = 0.0584, β = 0.0116, SE = 0.0061) were included, although heart diseases (p = 0.1015, β = 0.3338, SE = 0.2038) and lung diseases (p = 0.3638, β = 0.1839, SE = 0.2025) showed no association with smoking duration either (Supplementary Tables S14, S15).

**Table 3:** Symptoms related to mood associated with smoking in regression models.

	Smoking		
	Yes/no	Dosage	Duration
Depression			
n	5223	4151	861
p value	** < 1 × 10**^**–5**^	** < 1 × 10**^**–5**^	**0.0422**
β	0.3362	0.0199	0.0123
Anxiety			
n	5223	4152	861
p value	**2 × 10**^**–5**^	** < 1 × 10**^**–5**^	0.3799
β	0.2748	0.0182	0.0053
Dropped many activities and interests			
n	5205	4152	868
p value	** < 1 × 10**^**–5**^	** < 1 × 10**^**–5**^	** < 1 × 10**^**–5**^
β	0.3283	0.0165	0.0253
Life feels empty			
n	5201	4151	866
p value	**0.0004**	** < 1 × 10**^**–5**^	**0.0021**
β	0.2843	0.0181	0.0230
Getting bored often			
n	5207	4153	868
p value	** < 1 × 10**^**–5**^	** < 1 × 10**^**–5**^	** < 1 × 10**^**–5**^
β	0.4263	0.0187	0.0412
Being afraid something bad could happen			
n	5195	4147	864
p value	**0.0025**	**0.0002**	**0.0039**
β	0.2050	0.0126	0.0184
Feeling helpless often			
n	5193	4144	864
p value	**2 × 10**^**–5**^	**3 × 10**^**–5**^	**1 × 10**^**–5**^
β	0.3099	0.0151	0.0296
Prefer staying at home			
n	5196	4145	864
p value	**0.0007**	** < 1 × 10**^**–5**^	**0.0017**
β	0.1955	0.0150	0.0179
Feeling to have more memory problems than other people			
n	5206	4156	868
p value	**0.0019**	**0.0014**	**0.0038**
β	0.2027	0.0105	0.0178
Feeling pretty worthless			
n	5189	4138	862
p value	**0.0002**	** < 1 × 10**^**–5**^	** < 1 × 10**^**–5**^
β	0.2958	0.0185	0.0370
Feeling that situation is hopeless			
n	5184	4139	862
p value	** < 1 × 10**^**–5**^	**3 × 10**^**–5**^	**0.0001**
β	0.3846	0.0172	0.0304

p value (exploratory): multivariate regression to predict the respective symptoms related to mood adjusted for covariates by including AAE, gender, and disease duration (time between AAO and current age) in the model.

Significant values are in [bold].

## Discussion

In our exploratory analysis of the Fox Insight cohort, coffee drinking showed very little association with the severity of motor symptoms that we investigated in generalized linear models while adjusting for AAE, gender, and disease duration. Coffee drinking demonstrated a negative relationship with chewing and swallowing when coffee drinking was used as a binary yes–no indication, indicating fewer problems with chewing and swallowing when drinking coffee. Caffeine has previously been depicted to improve the motor deficits in PD or decelerate PD progression and ameliorate both motor and non-motor early symptoms^13,14^. There was a lack of association between non-motor symptoms and general coffee consumption as well as coffee intake duration in our study, however, we found a positive relationship between unexplained pains and the coffee drinking dosage. Nevertheless, the effect of coffee consumption and potential long-term effects need to be investigated in further confirmatory and longitudinal studies. Long-term effects of coffee drinking may positively impact memory, cognition, and verbal retrieval^6,15,16^.

We found an association between the general intake of aspirin and the number of pills per week with selected motor and non-motor symptoms, indicating more problems with the reported symptoms when using aspirin. In contrast, aspirin intake duration did not show an association with neither motor symptoms nor non-motor symptoms. Nevertheless, it is important to note that aspirin users are a heterogeneous group: the medication can be used for various reasons (e.g. anti-aggregation, cerebrovascular, and cardiovascular problems). Thus, we included heart diseases as an additional comorbidity in our models and the association with motor and non-motor features for aspirin were still robust. Nevertheless, the potential confounding effect of other comorbidities needs to be considered. The clinical effect of NSAIDs is still subject to controversial discussion. Although anti-inflammatory drugs such as aspirin and ibuprofen are known to reduce the risk for PD or delay PD onset^1,17–19^, the clinical impact on motor and non-motor symptom severity and progression remains unclear.

In addition, we found a positive association between the general smoking status, number of cigarettes per day, and smoking duration with selected motor symptoms. Smokers with PD exhibited more problems with speech, too much saliva, chewing and swallowing, walking and balance, freezing, and getting up with a different level of severity depending on the smoking dosage. The underlying cause of more severe motor symptoms in smokers remains unclear. Since smokers in this cohort were on average older than non-smokers but had a shorter disease duration (Supplementary Figures S2–S8), we would rather have expected less severe motor symptoms. In line with our findings, previous studies have reported later motor symptom onsets in smokers compared to non-smokers^20^, however, there was no report on more severe motor symptoms in smokers, but similar baseline motor deficits in smokers and non-smokers^21^. An excessive amount of saliva and swallowing dysfunctions resulting in drooling are clinically relevant symptoms in PD^22,23^, however, smokers have thicker saliva compared to non-smokers who tend to have predominantly serous saliva and the amount of saliva was found to decrease with the duration of smoking in the short term^24^. Interestingly, we observe that smokers with PD had increased saliva excess and drooling rather than decreased saliva in the long-term (mean disease duration of 6.5 years SD 5.7 years). There are also no reports on the effect of smoking on other bradykinetic symptoms like freezing or walking difficulties that would explain the increased problems in smokers. This further highlights the importance to investigate interactions between lifestyle factors and motor symptoms. When extending the investigations to non-motor symptoms, more problems with selected non-motor symptoms were observed when smoking. In addition, smoking correlated with symptoms related to depression and mood. A greater likelihood to experience feelings related to depression was reported when smoking or former smoking compared to never smoking. In general, there is evidence of an established relationship between smoking and mental health, showing that smoking increases the number of days with poor mental health, especially among individuals with more severe illnesses^25,26^. Still, the causal effect remains unclear. In other words, smoking itself may promote depression and anxiety, or patients with depression are just more likely to smoke and have greater difficulty quitting, which could indicate a reverse causation^27,28^. Reverse causation is a general risk that appears in cross-sectional studies. In addition, the results only show correlations but do not necessarily indicate causalities. To examine whether smoking has an actual impact on motor and non-motor symptoms, longitudinal studies need to be performed in the future to determine a possible long-lasting effect of tobacco use and smoking on PD-related symptoms. The impact of smoking should also be assessed for intake after AAO longitudinally.

One major strength of our study was the large sample size that provided sufficient power to assess lifestyle factors and clinical severity of motor and non-motor symptoms. The variability in age and age at onset did not affect our results after adjustment in our regression models. Although a mean AAO of 60.4 years is within the typical range for patients with PD^29^, the proportion of patients with early-onset PD (AAO < 50 years) is ~ 17%, slightly higher than expected^30^. Patients with an earlier PD onset might be more interested in participating in online studies. The same might also apply to individuals with a higher educational level. According to a study in 2020, the Fox Insight cohort has a greater educational attainment as compared to other cohorts^31^. When comparing the educational level in the different subgroups of lifestyle factors, we found smokers to have an overall lower educational level compared to non-smokers (Supplementary Figure S9). In addition, the smoking duration showed a trend of being shorter in the group of smokers with a higher educational level compared to smokers with a lower educational level. In contrast, there was a trend of a longer coffee drinking duration in the group of coffee drinkers with a higher educational level compared to coffee drinkers with a lower educational level (Supplementary Figure S10). There was no difference in the educational level between aspirin users and non-aspirin users (Supplementary Figure S11).

Additionally, as the Fox Insight study collects self-report data online, it offers many possibilities to promote epidemiological research. The convenience and accessibility for the participants and researchers allow easier patient recruitment and higher rates of return. This was previously investigated in a study by comparing the Fox Insight PD cohort's self-reported demographic characteristics, symptoms, medical history, and PD medication use to other in-person observational research study cohorts^31^. The authors found that patterns of responses to patient-reported assessments that were obtained online on the PD cohort of the Fox Insight study resembled PD cohorts assessed in person. In addition, patient-reported outcomes are becoming increasingly important to research, therapeutic development, and healthcare delivery, which was already investigated in another previous study on the Fox Insight cohort^32^.

However, we were limited to the questionnaires and data collected by Fox Insight in this study, including the selection of environmental factors and the types of questions. The motor and non-motor symptom scores were subjective evaluations and might differ from assessments by movement disorder specialists as the motor symptom questionnaire enquires about symptom severity over the past week. This way, it might reflect a personal snapshot of the current severity of symptoms but does not take possible off-episodes into account. Motor fluctuations are a major problem in advanced levodopa-treated PD patients, leading to “off” states, in which disability increases^33,34^. Adjusting for off-episodes in the regression models might be a crucial point to interpret the scores properly. Unfortunately, we were limited to the data collected by Fox Insight in this study and the numbers of PD patients responding to questionnaires about off-episodes were not sufficient to include in our analyses. In addition, it is important to mention that results were not corrected for multiple testing as analyses were not based on the presence of an ‘a priori’ hypothesis. Thus, they cannot be interpreted as significant after multiple testing correction and p values remain descriptive.

Nevertheless, these findings may help to acquire a better understanding of this complex disease. This study comprehensively assesses the effect of smoking, coffee drinking, and aspirin intake on clinical symptoms. These findings are so far only exploratory, however, they set the stage for future longitudinal assessments on these factors and PD clinical features.

## Supplementary Information

Supplementary Information.

## Data Availability

Data used in the preparation of this article were obtained from the Fox Insight database (https://foxinsight-info.michaeljfox.org/insight/explore/insight.jsp) on 18/10/2020. For up-to-date information on the study, visit https://foxinsight-info.michaeljfox.org/insight/explore/insight.jsp.
