# Supplementary material for: Lifestyle factors and clinical severity of Parkinson’s disease
Source: Sci Rep. 2023 Jun 12;13:9537. doi: 10.1038/s41598-023-31531-w (PMC10260917; doi:10.1038/s41598-023-31531-w)
Supplement: Supplementary file 1 — Supplementary Information. [file 41598_2023_31531_MOESM1_ESM.pdf]

**Supplementary Information:**

# Lifestyle factors and clinical severity of Parkinson’s disease

Carolin Gabbert[1], MSc, Prof. Inke R. König[2], Theresa Lüth[1], MSc, Meike Kasten[1,3], MD, Fox Insight Study[4], Anne Grünewald[1,5], PhD, Christine Klein[1], MD, Joanne Trinh[1*], PhD

[1]Institute of Neurogenetics, University of Lübeck, Lübeck, Germany
[2]Institute of Medical Biometry and Statistics, University of Lübeck, Lübeck, Germany
[3]Department of Psychiatry and Psychotherapy, University of Lübeck, Lübeck, Germany
[4]The Michael J. Fox Foundation for Parkinson’s Research, New York, NY, USA
[5]Luxembourg Centre for Systems Biomedicine, University of Luxembourg, Esch-sur-Alzette, Luxembourg

*** Correspondence:**
Joanne Trinh
University of Lübeck
Ratzeburger Allee 160
23538 Lübeck, Germany
Email: joanne.trinh@neuro.uni-luebeck.de
Tel.: +49-451-31018202

**Supplementary Table S1.** Demographics of the Fox Insight participants.

| Full study group (n=35,959) | Patients with PD |
|---|---|
| **Male (%)** | 18,349 (51.0%) |
| **Female (%)** | 14,528 (40.4%) |
| **Ethnicity:** | |
| **White/Caucasian (%)** | 32,332 (89.9%) |
| **Black/African American (%)** | 369 (1.0%) |
| **American Indian/Alaska Native (%)** | 393 (1.1%) |
| **Asian (%)** | 691 (1.9%) |
| **Native Hawaiian/Other Pacific Islander (%)** | 47 (0.1%) |
| **Hispanic/Latino/Spanish Origin (%)** | 1,692 (4.7%) |
| **Mean AAO (SD)** | 60.4 (11.0) |
| **Median AAO (IQR)** | 61.3 (53.6-68.1) |
| **Mean AAE (SD)** | 65.7 (10.2) |
| **Median AAE (IQR)** | 66.7 (59.6-72.6) |
| **Mean Current Age (SD)** | 66.9 (10.2) |
| **Median Current Age (IQR)** | 68.0 (60.8-73.7) |
| **Mean Disease Duration until Examination (SD)** | 5.3 (5.6) |
| **Median Disease Duration until Examination (IQR)** | 3.5 (1.2-7.6) |
| **Mean Disease Duration until Current Age (SD)** | 6.5 (5.7) |
| **Median Disease Duration until Current Age (IQR)** | 5.0 (2.5-8.9) |

**Supplementary Text:**

***Fox Insight study:***
The Fox Insight study is an ongoing online, longitudinal health study of people with and without PD with targeted enrollment set to at least 125,000 individuals[1]. The data is a rich data set facilitating discovery, validation, and reproducibility in PD research. The dataset is generated through routine longitudinal assessments (health and medical questionnaires evaluated at regular cycles); one-time health and disease questionnaires about symptoms, daily activities, and other factors; and, in a subgroup of people with PD, genetic data collection. Qualified researchers can explore, analyze, and download patient-reported outcomes (PROs) data and PD-related genetic variants at https://foxden.michaeljfox.org. The full Fox Insight genetic data set, including approximately 650,000 single nucleotide polymorphisms (SNPs) per participant, can be requested separately with institutional review.
Fox Insight participants were 18 years of age or older and provided informed consent. In the process of registration, participants were divided into two groups, PD patients and controls, the latter were asked about new diagnoses every three months. PD patients responded to health, non-motor assessments, motor assessments, quality of life, and lifestyle questionnaires. These questionnaires are based on the Movement Disorders Society – Unified Parkinson's disease Rating Scale (MDS-UPDRS) Part II, the Non-Motor Symptoms Questionnaire (NMSQ), and the Geriatric Depression Scale (GDS).
The PD-RFQ-U on "Smoking and Tobacco" questionnaire was used to evaluate smoking, the PD-RFQ-U on "Caffeine" to evaluate coffee drinking, and the PD-RFQ-U on "Anti-inflammatory Medication History" for anti-inflammatory drug intake. The surveys on "Your Movement Experiences" (MDS-UPDRS Part II; The scores range from 1 to 5, with higher scores indicating more severe symptoms) and "Your Non-Movement Experiences" (NMSQ; Scores of 0 and 1) were used to assess the association between smoking, coffee drinking, and aspirin intake with motor and non-motor symptoms. Finally, the surveys on "Your Current Health" and "Your Mood" (GDS; Scores of 0 and 1) were used to examine the association between smoking and mood, anxiety, and depression. All of these data were self-reported by the patients.
For each environmental or lifestyle factor, the corresponding datasets were downloaded from the FoxDEN website (https://foxden.michaeljfox.org/insight/explore/fox.jsp) (log:18/10/2020).

***Statistical analysis:***
We performed multilinear regression models to investigate the relationship between environmental factors, age, disease duration, and motor/non-motor symptoms (R studio).

*Regression model investigating environmental factors and motor/non-motor symptoms adjusted for AAE, gender, disease duration, and potential comorbidities (heart diseases and lung diseases for smoking, and heart diseases, arthritis, back pain, and surgeries with anesthesia for aspirin):*
→ glm(formula = MotorSymptomYes ~ AAE + Gender + DiseaseDuration (+ Comorbidity) + EnvFactorYes, family = binomial, data = data)
→ glm(formula = MotorSymptomYes ~ AAE + Gender + DiseaseDuration (+ Comorbidity) + EnvFactorDosage, family = binomial, data = data)
→ glm(formula = MotorSymptomYes ~ AAE + Gender + DiseaseDuration (+ Comorbidity) + EnvFactorDuration, family = binomial, data = data)
→ glm(formula = NonMotorSymptomYes ~ AAE + Gender + DiseaseDuration (+ Comorbidity) + EnvFactorYes, family = binomial, data = data)
→ glm(formula = NonMotorSymptomYes ~ AAE + Gender + DiseaseDuration (+ Comorbidity) + EnvFactorDosage, family = binomial, data = data)
→ glm(formula = NonMotorSymptomYes ~ AAE + Gender + DiseaseDuration (+ Comorbidity) + EnvFactorDuration, family = binomial, data = data)
→ glm(formula = MoodSymptomYes ~ AAE + Gender + DiseaseDuration (+ Comorbidity) + SmokingYes, family = binomial, data = data)
→ glm(formula = MoodSymptomYes ~ AAE + Gender + DiseaseDuration (+ Comorbidity) + SmokingDosage, family = binomial, data = data)

→ glm(formula = MoodSymptomYes ~ AAE + Gender + DiseaseDuration (+ Comorbidity) + SmokingDuration, family = binomial, data = data)

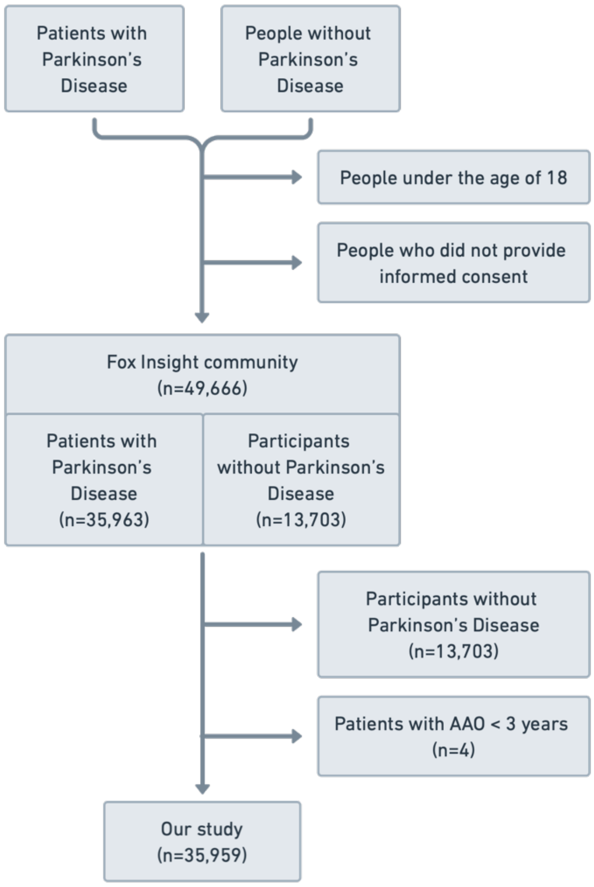


**Supplementary Figure S1.** Workflow of the inclusion and exclusion criteria of participant recruitment from the Fox Insight study and for this study.

**Supplementary Table S2.** Clinical variables.

| Your Movement Experiences | Your Non-Movement Experiences | Your Current Health | Your Mood |
|---|---|---|---|
| Tremor: Over the past week, have you usually had shaking or tremor? | Have you experienced constipation (less than three bowel movements a week) or having to strain to pass a stool in the last month? | Do you currently have depression? | Have you dropped many of your activities and interests? |
| Speech: Over the past week, have you had problems with your speech? | Have you experienced unexplained pains (not due to known conditions such as arthritis) in the last month? | Have you had anxiety? | Do you feel that your life is empty? |
| Saliva and Drooling: Over the past week, have you usually had too much saliva during when you are awake or when you sleep? | Have you experienced problems remembering things that have happened recently or forgetting to do things in the last month? | / | Do you often get bored? |
| Chewing and Swallowing: Over the past week, have you usually had problems swallowing pills or eating meals? Do you need your pills cut or crushed or your meals to be made soft, chopped or blended to avoid choking? | Have you experienced feeling sad, 'low' or 'blue' in the last month? | / | Are you afraid that something bad is going to happen to you? |
| Walking and Balance: Over the past week, have you usually had problems with balance and walking? | Have you experienced feeling anxious, frightened or panicky in the last month? | / | Do you often feel helpless? |
| Freezing: Over the past week, on your usual day when walking, do you suddenly stop or freeze as if your feet are stuck to the floor? | Have you experienced feeling less interested in sex or more interested in sex in the last month? | / | Do you prefer to stay at home, rather than going out and doing new things? |
| Getting out of bed, a care, or a deep chair: Over the past week, have you usually had trouble getting out of a bed, a car seat, or a deep chair? | Have you experienced feeling light-headed, dizzy or weak standing from sitting or lying in the last month? | / | Do you feel you have more problems with memory than most people? |
| / | / | / | Do you feel pretty worthless the way you are now? |
| / | / | / | Do you feel that your situation is hopeless? |

Motor and non-motor symptoms that were used from the surveys “Your Movement Experiences”, “Your Non-Movement Experiences”, “Your Current Health”, and “Your Mood”.

**Supplementary Table S3.** Generalized linear models on motor symptoms. Regression models for motor symptoms associated with coffee drinking, aspirin intake, and smoking in the Fox Insight cohort.

**Dependent variable: Chewing and Swallowing**

| Covariates | Estimate | Standard error | p-value |
|---|---|---|---|
| AAE | 0.0036 | 0.0034 | 0.2984 |
| Gender | -0.0193 | 0.0612 | 0.7521 |
| Disease Duration | 0.0735 | 0.0063 | $<1x10^{-5}$ |
| Coffee drinking (binary) | -0.1435 | 0.0731 | 0.0497 |

**Dependent variable: Tremor**

| Covariates | Estimate | Standard error | p-value |
|---|---|---|---|
| AAE | -0.0257 | 0.0057 | $<1x10^{-5}$ |
| Gender | -0.2811 | 0.0947 | 0.0030 |
| Disease Duration | -0.0307 | 0.0087 | 0.0004 |
| Aspirin intake (binary) | 0.3174 | 0.1054 | 0.0026 |

**Dependent variable: Chewing and Swallowing**

| Covariates | Estimate | Standard error | p-value |
|---|---|---|---|
| AAE | -0.0044 | 0.0047 | 0.3435 |
| Gender | -0.0027 | 0.0806 | 0.9733 |
| Disease Duration | 0.0722 | 0.0084 | $<1x10^{-5}$ |
| Aspirin intake (binary) | 0.1837 | 0.0875 | 0.0358 |

**Dependent variable: Getting Up**

| Covariates | Estimate | Standard error | p-value |
|---|---|---|---|
| AAE | 0.0215 | 0.0048 | $<1x10^{-5}$ |
| Gender | 0.0432 | 0.0832 | 0.6034 |
| Disease Duration | 0.1040 | 0.0109 | $<1x10^{-5}$ |
| Aspirin intake (binary) | 0.2170 | 0.0922 | 0.0185 |

**Dependent variable: Tremor**

| Covariates | Estimate | Standard error | p-value |
|---|---|---|---|
| AAE | -0.0249 | 0.0057 | $2x10^{-5}$ |
| Gender | -0.3021 | 0.0966 | 0.0018 |
| Disease Duration | -0.0329 | 0.0089 | 0.0002 |
| Aspirin intake Dosage | 0.0287 | 0.0117 | 0.0138 |

**Dependent variable: Chewing and Swallowing**

| Covariates | Estimate | Standard error | p-value |
|---|---|---|---|
| AAE | -0.0042 | 0.0048 | 0.3729 |
| Gender | -0.0326 | 0.0824 | 0.6927 |
| Disease Duration | 0.0736 | 0.0087 | $<1x10^{-5}$ |
| Aspirin intake Dosage | 0.0201 | 0.0085 | 0.0182 |

**Dependent variable: Walking and Balance**

| Covariates | Estimate | Standard error | p-value |
|---|---|---|---|
| AAE | 0.0024 | 0.0048 | 0.6178 |
| Gender | -0.0070 | 0.0846 | 0.9339 |
| Disease Duration | 0.1141 | 0.0114 | $<1x10^{-5}$ |
| Aspirin intake Dosage | 0.0253 | 0.0099 | 0.0106 |

**Dependent variable: Getting Up**

| Covariates | Estimate | Standard error | p-value |
|---|---|---|---|
| AAE | 0.0218 | 0.0048 | $<1x10^{-5}$ |
| Gender | 0.0565 | 0.0845 | 0.5039 |
| Disease Duration | 0.1038 | 0.0111 | $<1x10^{-5}$ |
| Aspirin intake Dosage | 0.0231 | 0.0098 | 0.0182 |

**Dependent variable: Saliva and Drooling**

| Covariates | Estimate | Standard error | p-value |
|---|---|---|---|
| AAE | 0.0114 | 0.0031 | 0.0003 |
| Gender | -0.5069 | 0.0563 | $<1x10^{-5}$ |
| Disease Duration | 0.0518 | 0.0059 | $<1x10^{-5}$ |
| Smoking (binary) | 0.1484 | 0.0580 | 0.0106 |

**Dependent variable: Chewing and Swallowing**

| Covariates | Estimate | Standard error | p-value |
|---|---|---|---|
| AAE | 0.0004 | 0.0033 | 0.9042 |
| Gender | -0.0550 | 0.0588 | 0.3495 |
| Disease Duration | 0.0706 | 0.0059 | $<1x10^{-5}$ |
| Smoking (binary) | 0.2243 | 0.0603 | 0.0002 |

**Dependent variable: Freezing**

| Covariates | Estimate | Standard error | p-value |
|---|---|---|---|
| AAE | 0.0006 | 0.0035 | 0.8747 |
| Gender | -0.0676 | 0.0630 | 0.2829 |
| Disease Duration | 0.1107 | 0.0064 | $<1x10^{-5}$ |
| Smoking (binary) | 0.1490 | 0.0646 | 0.0212 |

**Dependent variable: Speech**

| Covariates | Estimate | Standard error | p-value |
|---|---|---|---|
| AAE | -0.0017 | 0.0036 | 0.6296 |
| Gender | -0.5920 | 0.0649 | $<1x10^{-5}$ |
| Disease Duration | 0.0931 | 0.0076 | $<1x10^{-5}$ |
| Smoking Dosage | 0.0089 | 0.0033 | 0.0062 |

**Dependent variable: Saliva and Drooling**

| Covariates | Estimate | Standard error | p-value |
|---|---|---|---|
| AAE | 0.0092 | 0.0035 | 0.0085 |
| Gender | -0.5331 | 0.0632 | $<1x10^{-5}$ |
| Disease Duration | 0.0490 | 0.0066 | $<1x10^{-5}$ |
| Smoking Dosage | 0.0096 | 0.0031 | 0.0022 |

**Dependent variable: Chewing and Swallowing**

| Covariates | Estimate | Standard error | p-value |
|---|---|---|---|
| AAE | 0.0007 | 0.0037 | 0.8560 |
| Gender | 0.0369 | 0.0663 | 0.5778 |
| Disease Duration | 0.0721 | 0.0067 | $<1x10^{-5}$ |
| Smoking Dosage | 0.0174 | 0.0031 | $<1x10^{-5}$ |

**Dependent variable: Walking and Balance**

| Covariates | Estimate | Standard error | p-value |
|---|---|---|---|
| AAE | 0.0070 | 0.0037 | 0.0608 |
| Gender | 0.0507 | 0.0683 | 0.4577 |
| Disease Duration | 0.1015 | 0.0087 | $<1x10^{-5}$ |
| Smoking Dosage | 0.0101 | 0.0035 | 0.0038 |

**Dependent variable: Freezing**

| Covariates | Estimate | Standard error | p-value |
|---|---|---|---|
| AAE | -0.0012 | 0.0040 | 0.7601 |
| Gender | -0.0413 | 0.0714 | 0.5632 |
| Disease Duration | 0.1137 | 0.0073 | $<1x10^{-5}$ |
| Smoking Dosage | 0.0094 | 0.0034 | 0.0052 |

**Dependent variable: Getting Up**

| Covariates | Estimate | Standard error | p-value |
|---|---|---|---|
| AAE | 0.0240 | 0.0038 | $<1x10^{-5}$ |
| Gender | 0.0747 | 0.0690 | 0.2790 |
| Disease Duration | 0.0898 | 0.0086 | $<1x10^{-5}$ |
| Smoking Dosage | 0.0098 | 0.0036 | 0.0061 |

**Dependent variable: Speech**

| Covariates | Estimate | Standard error | p-value |
|---|---|---|---|
| AAE | -0.0068 | 0.0080 | 0.3913 |
| Gender | -0.7464 | 0.1449 | $<1x10^{-5}$ |
| Disease Duration | 0.0847 | 0.0186 | $<1x10^{-5}$ |
| Smoking Duration | 0.0119 | 0.0059 | 0.0454 |

**Dependent variable: Walking and Balance**

| Covariates | Estimate | Standard error | p-value |
|---|---|---|---|
| AAE | -0.0018 | 0.0083 | 0.8289 |
| Gender | 0.1699 | 0.1534 | 0.2682 |
| Disease Duration | 0.0981 | 0.0209 | $<1x10^{-5}$ |
| Smoking Duration | 0.0268 | 0.0065 | $4x10^{-5}$ |

**Dependent variable: Freezing**

| Covariates | Estimate | Standard error | p-value |
|---|---|---|---|
| AAE | -0.0150 | 0.0089 | 0.0918 |
| Gender | -0.1832 | 0.1603 | 0.2531 |
| Disease Duration | 0.0981 | 0.0168 | $<1x10^{-5}$ |
| Smoking Duration | 0.0277 | 0.0062 | $<1x10^{-5}$ |

**Dependent variable: Getting Up**

| Covariates | Estimate | Standard error | p-value |
|---|---|---|---|
| AAE | 0.0185 | 0.0084 | 0.0268 |
| Gender | 0.0647 | 0.1569 | 0.6801 |
| Disease Duration | 0.0851 | 0.0208 | $4x10^{-5}$ |
| Smoking Duration | 0.0366 | 0.0070 | $<1x10^{-5}$ |

**Supplementary Table S4.** Non-motor symptoms associated with environmental factors. Percentage of patients stratified by coffee consumption, aspirin intake, and smoking status and non-motor symptoms.

| | | Coffee | | | Aspirin | | | Smoking | | |
|---|---|---|---|---|---|---|---|---|---|---|
| | | yes | no | p-value | yes | no | p-value | yes | no | p-value |
| Constipation | yes | 53.5% (n=2127) | 57.0% (n=643) | 0.0970 | 55.2% (n=553) | 51.6% (n=1027) | **0.0124** | 55.1% (n=1174) | 53.6% (n=1801) | 0.1994 |
| | no | 46.5% (n=1847) | 43.0% (n=486) | | 44.8% (n=449) | 48.4% (n=962) | | 44.9% (n=957) | 46.4% (n=1559) | |
| Unexplained Pains | yes | 37.2% (n=1480) | 37.5% (n=423) | 0.1623 | 37.8% (n=379) | 38.5% (n=765) | **0.0227** | 40.0% (n=852) | 35.7% (n=1199) | **$<1x10^{-5}$** |
| | no | 62.8% (n=2494) | 62.5% (n=706) | | 62.2% (n=623) | 61.5% (n=1224) | | 60.0% (n=1278) | 64.3% (n=2161) | |
| Problems Remembering | yes | 47.0% (n=1869) | 46.8% (n=528) | 0.9859 | 50.1% (n=502) | 41.9% (n=834) | **$1x10^{-5}$** | 50.6% (n=1078) | 44.9% (n=1510) | **0.0001** |
| | no | 53.0% (n=2105) | 53.2% (n=601) | | 49.9% (n=500) | 58.1% (n=1155) | | 49.4% (n=1052) | 55.1% (n=1850) | |
| Feeling Sad | yes | 50.2% (n=1993) | 50.3% (n=567) | 0.2517 | 47.9% (n=480) | 50.1% (n=995) | 0.0665 | 53.9% (n=1148) | 48.0% (n=1611) | **$<1x10^{-5}$** |
| | no | 49.8% (n=1980) | 49.7% (n=560) | | 52.1% (n=522) | 49.9% (n=993) | | 46.1% (n=982) | 52.0% (n=1746) | |
| Anxiety | yes | 36.2% (n=1438) | 35.9% (n=405) | 0.2199 | 32.9% (n=330) | 37.2% (n=740) | 0.2999 | 38.7% (n=825) | 34.2% (n=1149) | **$<1x10^{-5}$** |
| | no | 63.8% (n=2535) | 64.1% (n=722) | | 67.1% (n=672) | 62.8% (n=1248) | | 61.3% (n=1305) | 65.8% (n=2208) | |
| Changed Interest in Sex | yes | 34.6% (n=1376) | 32.3% (n=364) | 0.1032 | 34.9% (n=350) | 32.6% (n=649) | **0.0221** | 35.7% (n=761) | 32.4% (n=1089) | **0.0013** |
| | no | 65.4% (n=2597) | 67.7% (n=763) | | 65.1% (n=652) | 67.4% (n=1339) | | 64.3% (n=1369) | 67.6% (n=2268) | |
| Light-headedness | yes | 43.4% (n=1723) | 41.7% (n=470) | 0.2808 | 45.1% (n=452) | 40.7% (n=810) | **0.0043** | 45.7% (n=974) | 41.4% (n=1390) | **0.0005** |
| | no | 56.6% (n=2250) | 58.3% (n=656) | | 54.9% (n=550) | 59.3% (n=1178) | | 54.3% (n=1156) | 58.6% (n=1966) | |

P-value (exploratory): Multivariate regression to predict the respective non-motor symptoms adjusted for covariates by including AAE, gender, and disease duration (time between AAO and current age).

**Supplementary Table S5.** Generalized linear models on non-motor symptoms. Regression models for non-motor symptoms associated with coffee drinking, aspirin intake, and smoking in the Fox Insight cohort.

**Dependent variable: Unexplained Pains**

| Covariates | Estimate | Standard error | p-value |
|---|---|---|---|
| AAE | -0.0305 | 0.0038 | $<1x10^{-5}$ |
| Gender | 0.4508 | 0.0687 | $<1x10^{-5}$ |
| Disease Duration | 0.0118 | 0.0066 | 0.0749 |
| Coffee drinking Dosage | 0.0083 | 0.0035 | 0.0168 |

**Dependent variable: Constipation**

| Covariates | Estimate | Standard error | p-value |
|---|---|---|---|
| AAE | -0.0003 | 0.0044 | 0.9542 |
| Gender | 0.0580 | 0.0759 | 0.4447 |
| Disease Duration | 0.0315 | 0.0079 | $7x10^{-5}$ |
| Aspirin intake (binary) | 0.2077 | 0.0831 | 0.0124 |

**Dependent variable: Unexplained Pains**

| Covariates | Estimate | Standard error | p-value |
|---|---|---|---|
| AAE | -0.0302 | 0.0046 | $<1x10^{-5}$ |
| Gender | 0.3524 | 0.0786 | $<1x10^{-5}$ |
| Disease Duration | 0.0181 | 0.0077 | 0.0188 |
| Aspirin intake (binary) | 0.1961 | 0.0861 | 0.0227 |

**Dependent variable: Problems Remembering**

| Covariates | Estimate | Standard error | p-value |
|---|---|---|---|
| AAE | -0.0065 | 0.0044 | 0.1399 |
| Gender | -0.1202 | 0.0763 | 0.1150 |
| Disease Duration | 0.0129 | 0.0076 | 0.0894 |
| Aspirin intake (binary) | 0.3662 | 0.0830 | $1x10^{-5}$ |

**Dependent variable: Changed Interest in Sex**

| Covariates | Estimate | Standard error | p-value |
|---|---|---|---|
| AAE | -0.0338 | 0.0047 | $<1x10^{-5}$ |
| Gender | -0.5160 | 0.0817 | $<1x10^{-5}$ |
| Disease Duration | 0.0187 | 0.0079 | 0.0179 |
| Aspirin intake (binary) | 0.2023 | 0.0884 | 0.0221 |

**Dependent variable: Light-headedness**

| Covariates | Estimate | Standard error | p-value |
|---|---|---|---|
| AAE | -0.0117 | 0.0044 | 0.0085 |
| Gender | 0.0299 | 0.0766 | 0.6960 |
| Disease Duration | 0.0013 | 0.0076 | 0.8615 |
| Aspirin intake (binary) | 0.2380 | 0.0833 | 0.0043 |

**Dependent variable: Constipation**

| Covariates | Estimate | Standard error | p-value |
|---|---|---|---|
| AAE | 0.0021 | 0.0044 | 0.6421 |
| Gender | 0.0674 | 0.0775 | 0.3846 |
| Disease Duration | 0.0316 | 0.0082 | 0.0001 |
| Aspirin intake Dosage | 0.0251 | 0.0086 | 0.0037 |

**Dependent variable: Unexplained Pains**

| Covariates | Estimate | Standard error | p-value |
|---|---|---|---|
| AAE | -0.0298 | 0.0046 | $<1x10^{-5}$ |
| Gender | 0.3633 | 0.0804 | $<1x10^{-5}$ |
| Disease Duration | 0.0191 | 0.0080 | 0.0163 |
| Aspirin intake Dosage | 0.0178 | 0.0083 | 0.0320 |

**Dependent variable: Problems Remembering**

| Covariates | Estimate | Standard error | p-value |
|---|---|---|---|
| AAE | -0.0054 | 0.0045 | 0.2262 |
| Gender | -0.1140 | 0.0779 | 0.1432 |
| Disease Duration | 0.0133 | 0.0078 | 0.0887 |
| Aspirin intake Dosage | 0.0295 | 0.0085 | 0.0005 |

**Dependent variable: Feeling Sad**

| Covariates | Estimate | Standard error | p-value |
|---|---|---|---|
| AAE | -0.0354 | 0.0046 | $<1x10^{-5}$ |
| Gender | 0.3975 | 0.0783 | $<1x10^{-5}$ |
| Disease Duration | 0.0168 | 0.0079 | 0.0331 |
| Aspirin intake Dosage | 0.0177 | 0.0083 | 0.0344 |

**Dependent variable: Changed Interest in Sex**

| Covariates | Estimate | Standard error | p-value |
|---|---|---|---|
| AAE | -0.0321 | 0.0048 | $<1x10^{-5}$ |
| Gender | -0.5400 | 0.0836 | $<1x10^{-5}$ |
| Disease Duration | 0.0171 | 0.0082 | 0.0358 |
| Aspirin intake Dosage | 0.0184 | 0.0084 | 0.0278 |

**Dependent variable: Light-headedness**

| Covariates | Estimate | Standard error | p-value |
|---|---|---|---|
| AAE | -0.0108 | 0.0045 | 0.0157 |
| Gender | 0.0416 | 0.0782 | 0.5949 |
| Disease Duration | 0.0035 | 0.0078 | 0.6538 |
| Aspirin intake Dosage | 0.0226 | 0.0082 | 0.0060 |

**Dependent variable: Unexplained Pains**

| Covariates | Estimate | Standard error | p-value |
|---|---|---|---|
| AAE | -0.0320 | 0.0032 | $<1x10^{-5}$ |
| Gender | 0.3859 | 0.0579 | $<1x10^{-5}$ |
| Disease Duration | 0.0149 | 0.0055 | 0.0069 |
| Smoking (binary) | 0.2732 | 0.0595 | $<1x10^{-5}$ |

**Dependent variable: Problems Remembering**

| Covariates | Estimate | Standard error | p-value |
|---|---|---|---|
| AAE | 0.0018 | 0.0031 | 0.5498 |
| Gender | -0.2018 | 0.0555 | 0.0003 |
| Disease Duration | 0.0155 | 0.0054 | 0.0039 |
| Smoking (binary) | 0.2176 | 0.0570 | 0.0001 |

**Dependent variable: Feeling Sad**

| Covariates | Estimate | Standard error | p-value |
|---|---|---|---|
| AAE | -0.0299 | 0.0032 | $<1x10^{-5}$ |
| Gender | 0.3498 | 0.0560 | $<1x10^{-5}$ |
| Disease Duration | 0.0139 | 0.0054 | 0.0100 |
| Smoking (binary) | 0.3279 | 0.0579 | $<1x10^{-5}$ |

**Dependent variable: Anxiety**

| Covariates | Estimate | Standard error | p-value |
|---|---|---|---|
| AAE | -0.0395 | 0.0033 | $<1x10^{-5}$ |
| Gender | 0.3907 | 0.0587 | $<1x10^{-5}$ |
| Disease Duration | -0.0005 | 0.0057 | 0.9244 |
| Smoking (binary) | 0.3007 | 0.0604 | $<1x10^{-5}$ |

**Dependent variable: Changed Interest in Sex**

| Covariates | Estimate | Standard error | p-value |
|---|---|---|---|
| AAE | -0.0335 | 0.0033 | $<1x10^{-5}$ |
| Gender | -0.5699 | 0.0600 | $<1x10^{-5}$ |
| Disease Duration | 0.0175 | 0.0056 | 0.0019 |
| Smoking (binary) | 0.1959 | 0.0610 | 0.0013 |

**Dependent variable: Light-headedness**

| Covariates | Estimate | Standard error | p-value |
|---|---|---|---|
| AAE | -0.0124 | 0.0031 | $6x10^{-5}$ |
| Gender | 0.0706 | 0.0558 | 0.2062 |
| Disease Duration | 0.0004 | 0.0054 | 0.9412 |
| Smoking (binary) | 0.2000 | 0.0574 | 0.0005 |

**Dependent variable: Unexplained Pains**

| Covariates | Estimate | Standard error | p-value |
|---|---|---|---|
| AAE | -0.0320 | 0.0036 | $<1x10^{-5}$ |
| Gender | 0.4477 | 0.0653 | $<1x10^{-5}$ |
| Disease Duration | 0.0136 | 0.0063 | 0.0306 |
| Smoking Dosage | 0.0114 | 0.0031 | 0.0003 |

**Dependent variable: Problems Remembering**

| Covariates | Estimate | Standard error | p-value |
|---|---|---|---|
| AAE | -0.0002 | 0.0034 | 0.9481 |
| Gender | -0.0999 | 0.0624 | 0.1091 |
| Disease Duration | 0.0189 | 0.0061 | 0.0020 |
| Smoking Dosage | 0.0123 | 0.0030 | $6x10^{-5}$ |

**Dependent variable: Feeling Sad**

| Covariates | Estimate | Standard error | p-value |
|---|---|---|---|
| AAE | -0.0291 | 0.0035 | $<1\text{x}10^{-5}$ |
| Gender | 0.3590 | 0.0629 | $<1\text{x}10^{-5}$ |
| Disease Duration | 0.0134 | 0.0061 | 0.0282 |
| Smoking Dosage | 0.0152 | 0.0031 | $<1\text{x}10^{-5}$ |

**Dependent variable: Anxiety**

| Covariates | Estimate | Standard error | p-value |
|---|---|---|---|
| AAE | -0.0377 | 0.0037 | $<1\text{x}10^{-5}$ |
| Gender | 0.3896 | 0.0662 | $<1\text{x}10^{-5}$ |
| Disease Duration | -0.0008 | 0.0065 | 0.9022 |
| Smoking Dosage | 0.0161 | 0.0031 | $<1\text{x}10^{-5}$ |

**Dependent variable: Light-headedness**

| Covariates | Estimate | Standard error | p-value |
|---|---|---|---|
| AAE | -0.0115 | 0.0035 | 0.0009 |
| Gender | 0.1096 | 0.0629 | 0.0812 |
| Disease Duration | -0.0002 | 0.0061 | 0.9740 |
| Smoking Dosage | 0.0117 | 0.0030 | 0.0001 |

**Dependent variable: Unexplained Pains**

| Covariates | Estimate | Standard error | p-value |
|---|---|---|---|
| AAE | -0.0482 | 0.0081 | $<1\text{x}10^{-5}$ |
| Gender | 0.2455 | 0.1420 | 0.0838 |
| Disease Duration | 0.0167 | 0.0144 | 0.2476 |
| Smoking Duration | 0.0140 | 0.0057 | 0.0134 |

**Dependent variable: Problems Remembering**

| Covariates | Estimate | Standard error | p-value |
|---|---|---|---|
| AAE | -0.0048 | 0.0076 | 0.5312 |
| Gender | -0.4815 | 0.1392 | 0.0005 |
| Disease Duration | -0.0150 | 0.0143 | 0.2951 |
| Smoking Duration | 0.0176 | 0.0056 | 0.0017 |

**Dependent variable: Feeling Sad**

| Covariates | Estimate | Standard error | p-value |
|---|---|---|---|
| AAE | -0.0460 | 0.0082 | $<1x10^{-5}$ |
| Gender | 0.2244 | 0.1407 | 0.1107 |
| Disease Duration | 0.0024 | 0.0143 | 0.8691 |
| Smoking Duration | 0.0112 | 0.0056 | 0.0470 |

**Dependent variable: Changed Interest in Sex**

| Covariates | Estimate | Standard error | p-value |
|---|---|---|---|
| AAE | -0.0451 | 0.0082 | $<1x10^{-5}$ |
| Gender | -0.7188 | 0.1524 | $<1x10^{-5}$ |
| Disease Duration | 0.0249 | 0.0147 | 0.0901 |
| Smoking Duration | 0.0123 | 0.0059 | 0.0372 |

**Supplementary Table S6.** Motor symptoms associated with aspirin intake in regression models including potential comorbidities.

| | | **Heart Diseases** | | | **Arthritis** | | | **Back Pain** | | | **Surgeries with Anesthesia** | | |
|---|---|---|---|---|---|---|---|---|---|---|---|---|---|
| | | **Aspirin** | | | | | | | | | | | |
| | | Yes/No | Dosage | Duration | Yes/No | Dosage | Duration | Yes/No | Dosage | Duration | Yes/No | Dosage | Duration |
| Tremor | n | 2854 | 2718 | 546 | 2849 | 2713 | 545 | 2850 | 2714 | 546 | 2852 | 2716 | 546 |
| | p-value | **0.0040** | **0.0212** | 0.5592 | **0.0031** | **0.0163** | 0.5430 | **0.0045** | **0.0210** | 0.5687 | **0.0029** | **0.0149** | 0.5788 |
| | β | 0.3098 | 0.0270 | 0.0063 | 0.3123 | 0.0281 | 0.0066 | 0.3007 | 0.0268 | 0.0061 | 0.3149 | 0.0284 | 0.0060 |
| Speech | n | 2856 | 2720 | 546 | 2851 | 2715 | 545 | 2852 | 2716 | 546 | 2854 | 2718 | 546 |
| | p-value | 0.4252 | 0.1185 | 0.3991 | 0.4522 | 0.1143 | 0.3428 | 0.4883 | 0.1146 | 0.3807 | 0.4293 | 0.1144 | 0.2840 |
| | β | -0.0704 | 0.0141 | -0.0069 | -0.0653 | 0.0141 | -0.0078 | -0.0601 | 0.0141 | -0.0072 | -0.0687 | 0.0141 | -0.0088 |
| Saliva and Drooling | n | 2856 | 2720 | 546 | 2851 | 2715 | 545 | 2852 | 2716 | 546 | 2854 | 2718 | 546 |
| | p-value | 0.3411 | 0.4267 | 0.5394 | 0.2549 | 0.3234 | 0.5455 | 0.2677 | 0.3498 | 0.5707 | 0.2371 | 0.2968 | 0.5351 |
| | β | 0.0816 | 0.0066 | 0.0049 | 0.0959 | 0.0082 | 0.0049 | 0.0933 | 0.0077 | 0.0046 | 0.0996 | 0.0087 | 0.0050 |
| Chewing and Swallowing | n | 2856 | 2720 | 546 | 2851 | 2715 | 545 | 2852 | 2716 | 546 | 2854 | 2718 | 546 |
| | p-value | 0.1120 | **0.0374** | 0.9540 | 0.0556 | **0.0287** | 0.8214 | 0.0591 | **0.0331** | 0.8354 | 0.0591 | **0.0256** | 0.8129 |
| | β | 0.1425 | 0.0180 | -0.0005 | 0.1684 | 0.0188 | -0.0019 | 0.1661 | 0.0182 | -0.0018 | 0.1661 | 0.0191 | -0.0020 |
| Walking and Balance | n | 2854 | 2718 | 546 | 2849 | 2713 | 545 | 2850 | 2714 | 546 | 2852 | 2716 | 546 |
| | p-value | 0.3443 | **0.0330** | 0.8998 | 0.1785 | **0.0211** | 0.7816 | 0.1729 | **0.0280** | 0.7627 | 0.1074 | **0.0086** | 0.7662 |
| | β | 0.0883 | 0.0212 | -0.0011 | 0.1246 | 0.0233 | -0.0024 | 0.1264 | 0.0223 | -0.0027 | 0.1477 | 0.0263 | -0.0026 |
| Freezing | n | 2854 | 2718 | 546 | 2849 | 2713 | 545 | 2850 | 2714 | 546 | 2852 | 2716 | 546 |
| | p-value | 0.3930 | 0.1566 | 0.8882 | 0.4965 | 0.2760 | 0.9973 | 0.3731 | 0.2004 | 0.9827 | 0.3905 | 0.1797 | 0.9949 |
| | β | 0.0836 | 0.0132 | 0.0013 | 0.0657 | 0.0101 | $-3\times10^{-5}$ | 0.0857 | 0.0118 | 0.0002 | 0.0826 | 0.0124 | $-6\times10^{-5}$ |
| Getting up | n | 2854 | 2718 | 546 | 2849 | 2713 | 545 | 2850 | 2714 | 546 | 2852 | 2716 | 546 |
| | p-value | 0.0534 | **0.0308** | 0.3426 | **0.0391** | **0.0367** | 0.3113 | **0.0375** | 0.0509 | 0.2534 | **0.0252** | **0.0188** | 0.2057 |
| | β | 0.1819 | 0.0214 | -0.0081 | 0.1924 | 0.0209 | -0.0087 | 0.1950 | 0.0196 | -0.0099 | 0.2074 | 0.0233 | -0.0110 |

P-value (exploratory): Multivariate regression to predict the respective motor symptoms adjusted for covariates by including AAE, gender, disease duration (time between AAO and current age), and comorbidities in the model.

**Supplementary Table S7.** Generalized linear models on motor symptoms including potential comorbidities. Regression models for motor symptoms with a change in outcome for aspirin intake and smoking in the Fox Insight cohort while adjusting for potential comorbidities.

**Dependent variable: Chewing and Swallowing**

| Covariates | Estimate | Standard error | p-value |
|---|---|---|---|
| AAE | -0.0055 | 0.0048 | 0.2485 |
| Gender | 0.0084 | 0.0811 | 0.9178 |
| Disease Duration | 0.0729 | 0.0085 | $<1x10^{-5}$ |
| Heart Diseases (binary) | 0.2666 | 0.1173 | 0.0231 |
| Aspirin intake (binary) | 0.1425 | 0.0897 | 0.1120 |

**Dependent variable: Chewing and Swallowing**

| Covariates | Estimate | Standard error | p-value |
|---|---|---|---|
| AAE | -0.0061 | 0.0048 | 0.2068 |
| Gender | -0.0500 | 0.0823 | 0.5437 |
| Disease Duration | 0.0724 | 0.0085 | $<1x10^{-5}$ |
| Arthritis (binary) | 0.2401 | 0.0833 | 0.0040 |
| Aspirin intake (binary) | 0.1684 | 0.0880 | 0.0556 |

**Dependent variable: Chewing and Swallowing**

| Covariates | Estimate | Standard error | p-value |
|---|---|---|---|
| AAE | -0.0049 | 0.0047 | 0.3007 |
| Gender | -0.0042 | 0.0811 | 0.9582 |
| Disease Duration | 0.0708 | 0.0085 | $<1x10^{-5}$ |
| Back Pain (binary) | 0.3360 | 0.0805 | $3x10^{-5}$ |
| Aspirin intake (binary) | 0.1661 | 0.0880 | 0.0591 |

**Dependent variable: Chewing and Swallowing**

| Covariates | Estimate | Standard error | p-value |
|---|---|---|---|
| AAE | -0.0055 | 0.0048 | 0.2487 |
| Gender | -0.0278 | 0.0814 | 0.7328 |
| Disease Duration | 0.0725 | 0.0084 | $<1x10^{-5}$ |
| Surgeries with Anesthesia (binary) | 0.4380 | 0.1503 | 0.0036 |
| Aspirin intake (binary) | 0.1661 | 0.0880 | 0.0591 |

**Dependent variable: Getting Up**

| Covariates | Estimate | Standard error | p-value |
|---|---|---|---|
| AAE | 0.0202 | 0.0048 | $3x10^{-5}$ |
| Gender | 0.0546 | 0.0835 | 0.5289 |
| Disease Duration | 0.1039 | 0.0109 | $<1x10^{-5}$ |
| Heart Diseases (binary) | 0.2775 | 0.1300 | 0.0328 |
| Aspirin intake (binary) | 0.1819 | 0.0941 | 0.0534 |

**Dependent variable: Getting Up**

| Covariates | Estimate | Standard error | p-value |
|---|---|---|---|
| AAE | 0.0202 | 0.0049 | $4x10^{-5}$ |
| Gender | 0.0397 | 0.0858 | 0.6433 |
| Disease Duration | 0.1006 | 0.0112 | $<1x10^{-5}$ |
| Back Pain (binary) | 0.7012 | 0.0858 | $<1x10^{-5}$ |
| Aspirin intake Dosage | 0.0196 | 0.0100 | 0.0509 |

**Dependent variable: Speech**

| Covariates | Estimate | Standard error | p-value |
|---|---|---|---|
| AAE | -0.0079 | 0.0082 | 0.3311 |
| Gender | -0.7369 | 0.1458 | $<1x10^{-5}$ |
| Disease Duration | 0.0841 | 0.0186 | $<1x10^{-5}$ |
| Heart Diseases (binary) | 0.0752 | 0.2002 | 0.7074 |
| Smoking Duration | 0.0115 | 0.0059 | 0.0526 |

**Dependent variable: Speech**

| Covariates | Estimate | Standard error | p-value |
|---|---|---|---|
| AAE | -0.0074 | 0.0080 | 0.3550 |
| Gender | -0.7417 | 0.1451 | $<1x10^{-5}$ |
| Disease Duration | 0.0839 | 0.0186 | $<1x10^{-5}$ |
| Lung Diseases (binary) | 0.1105 | 0.2011 | 0.5828 |
| Smoking Duration | 0.0113 | 0.0060 | 0.0591 |

**Supplementary Table S8.** Non-motor symptoms associated with aspirin intake in regression models including potential comorbidities.

| | | Heart Diseases | | | Arthritis | | | Back Pain | | | Surgeries with Anesthesia | | |
|---|---|---|---|---|---|---|---|---|---|---|---|---|---|
| | | Aspirin | | | | | | | | | | | |
| | | Yes/No | Dosage | Duration | Yes/No | Dosage | Duration | Yes/No | Dosage | Duration | Yes/No | Dosage | Duration |
| Constipation | n | 2865 | 2727 | 547 | 2860 | 2722 | 546 | 2861 | 2723 | 547 | 2863 | 2725 | 547 |
| | p-value | **0.0264** | **0.0055** | 0.6126 | **0.0136** | **0.0040** | 0.6505 | **0.0200** | **0.0083** | 0.7533 | **0.0144** | **0.0036** | 0.6749 |
| | β | 0.1888 | 0.0244 | 0.0041 | 0.2061 | 0.0252 | 0.0036 | 0.1954 | 0.0231 | 0.0026 | 0.2044 | 0.0254 | 0.0034 |
| Unexplained Pains | n | 2865 | 2727 | 547 | 2860 | 2722 | 546 | 2861 | 2723 | 547 | 2863 | 2725 | 547 |
| | p-value | **0.0220** | **0.0338** | 0.7971 | **0.0383** | 0.0672 | 0.7426 | **0.0414** | 0.0896 | 0.6581 | **0.0275** | **0.0419** | 0.7705 |
| | β | 0.2020 | 0.0179 | -0.0022 | 0.1799 | 0.0154 | -0.0028 | 0.1788 | 0.0142 | -0.0038 | 0.1909 | 0.0170 | -0.0024 |
| Problems Remembering | n | 2865 | 2727 | 547 | 2860 | 2722 | 546 | 2861 | 2723 | 547 | 2863 | 2725 | 547 |
| | p-value | **0.0003** | **0.0033** | 0.6288 | **$2x10^{-5}$** | **0.0012** | 0.7542 | **$2x10^{-5}$** | **0.0014** | 0.7882 | **$3x10^{-5}$** | **0.0011** | 0.7528 |
| | β | 0.3080 | 0.0249 | 0.0038 | 0.3545 | 0.0276 | 0.0025 | 0.3554 | 0.0271 | 0.0021 | 0.3481 | 0.0278 | 0.0025 |
| Feeling Sad | n | 2865 | 2727 | 547 | 2860 | 2722 | 546 | 2861 | 2723 | 547 | 2863 | 2725 | 547 |
| | p-value | 0.2404 | 0.0806 | 0.3990 | 0.1073 | 0.0513 | 0.4817 | 0.0946 | 0.0527 | 0.4985 | 0.0807 | **0.0350** | 0.4154 |
| | β | 0.1009 | 0.0147 | 0.0068 | 0.1360 | 0.0164 | 0.0057 | 0.1414 | 0.0163 | 0.0055 | 0.1472 | 0.0177 | 0.0066 |
| Anxiety | n | 2865 | 2727 | 547 | 2860 | 2722 | 546 | 2861 | 2723 | 547 | 2863 | 2725 | 547 |
| | p-value | 0.5224 | 0.2396 | 0.9046 | 0.3557 | 0.1719 | 0.8528 | 0.3786 | 0.2036 | 0.8032 | 0.2894 | 0.1349 | 0.8282 |
| | β | 0.0582 | 0.0100 | -0.0010 | 0.0824 | 0.0115 | -0.0016 | 0.0787 | 0.0107 | -0.0021 | 0.0946 | 0.0126 | -0.0019 |
| Changed Interest in Sex | n | 2865 | 2727 | 547 | 2860 | 2722 | 546 | 2861 | 2723 | 547 | 2863 | 2725 | 547 |
| | p-value | **0.0379** | **0.0309** | 0.7100 | **0.0178** | **0.0185** | 0.6927 | **0.0266** | **0.0310** | 0.7401 | **0.0210** | **0.0223** | 0.6549 |
| | β | 0.1882 | 0.0184 | 0.0032 | 0.2105 | 0.0199 | 0.0034 | 0.1968 | 0.0182 | 0.0028 | 0.2051 | 0.0193 | 0.0038 |
| Light-headedness | n | 2865 | 2727 | 547 | 2860 | 2722 | 546 | 2861 | 2723 | 547 | 2863 | 2725 | 547 |
| | p-value | **0.0396** | **0.0296** | 0.5597 | **0.0083** | **0.0130** | 0.6834 | **0.0092** | **0.0161** | 0.6425 | **0.0084** | **0.0108** | 0.6331 |
| | β | 0.1761 | 0.0180 | 0.0046 | 0.2218 | 0.0206 | 0.0033 | 0.2193 | 0.0198 | 0.0037 | 0.2211 | 0.0210 | 0.0038 |

P-value (exploratory): Multivariate regression to predict the respective non-motor symptoms adjusted for covariates by including AAE, gender, disease duration (time between AAO and current age), and comorbidities in the model.

**Supplementary Table S9.** Generalized linear models on non-motor symptoms including potential comorbidities. Regression models for non-motor symptoms with a change in outcome for aspirin intake and smoking in the Fox Insight cohort while adjusting for potential comorbidities.

**Dependent variable: Unexplained Pains**

| Covariates | Estimate | Standard error | p-value |
|---|---|---|---|
| AAE | -0.0343 | 0.0048 | $<1x10^{-5}$ |
| Gender | 0.3041 | 0.0822 | 0.0002 |
| Disease Duration | 0.0195 | 0.0081 | 0.0156 |
| Arthritis (binary) | 0.3817 | 0.0842 | $<1x10^{-5}$ |
| Aspirin intake Dosage | 0.0154 | 0.0084 | 0.0672 |

**Dependent variable: Unexplained Pains**

| Covariates | Estimate | Standard error | p-value |
|---|---|---|---|
| AAE | -0.0332 | 0.0048 | $<1x10^{-5}$ |
| Gender | 0.3706 | 0.0820 | $<1x10^{-5}$ |
| Disease Duration | 0.0159 | 0.0082 | 0.0516 |
| Back Pain (binary) | 0.7359 | 0.0825 | $<1x10^{-5}$ |
| Aspirin intake Dosage | 0.0142 | 0.0084 | 0.0896 |

**Dependent variable: Feeling Sad**

| Covariates | Estimate | Standard error | p-value |
|---|---|---|---|
| AAE | -0.0385 | 0.0047 | $<1x10^{-5}$ |
| Gender | 0.4128 | 0.0790 | $<1x10^{-5}$ |
| Disease Duration | 0.0170 | 0.0079 | 0.0327 |
| Heart Diseases (binary) | 0.3383 | 0.1187 | 0.0044 |
| Aspirin intake Dosage | 0.0147 | 0.0084 | 0.0806 |

**Dependent variable: Feeling Sad**

| Covariates | Estimate | Standard error | p-value |
|---|---|---|---|
| AAE | -0.0399 | 0.0047 | $<1x10^{-5}$ |
| Gender | 0.3368 | 0.0799 | $3x10^{-5}$ |
| Disease Duration | 0.0161 | 0.0080 | 0.0430 |
| Arthritis (binary) | 0.3183 | 0.0821 | 0.0001 |
| Aspirin intake Dosage | 0.0164 | 0.0084 | 0.0513 |

**Dependent variable: Feeling Sad**

| Covariates | Estimate | Standard error | p-value |
|---|---|---|---|
| AAE | -0.0382 | 0.0047 | $<1x10^{-5}$ |
| Gender | 0.3930 | 0.0790 | $<1x10^{-5}$ |
| Disease Duration | 0.0133 | 0.0080 | 0.0955 |
| Back Pain (binary) | 0.4246 | 0.0790 | $<1x10^{-5}$ |
| Aspirin intake Dosage | 0.0163 | 0.0084 | 0.0527 |

**Dependent variable: Feeling Sad**

| Covariates | Estimate | Standard error | p-value |
|---|---|---|---|
| AAE | -0.0497 | 0.0085 | $<1x10^{-5}$ |
| Gender | 0.2623 | 0.1422 | 0.0650 |
| Disease Duration | 0.0015 | 0.0143 | 0.9170 |
| Heart Diseases (binary) | 0.4377 | 0.1920 | 0.0226 |
| Smoking Duration | 0.0099 | 0.0057 | 0.0807 |

**Dependent variable: Feeling Sad**

| Covariates | Estimate | Standard error | p-value |
|---|---|---|---|
| AAE | -0.0464 | 0.0083 | $<1x10^{-5}$ |
| Gender | 0.2334 | 0.1412 | 0.0985 |
| Disease Duration | 0.0016 | 0.0144 | 0.9112 |
| Lung Diseases (binary) | 0.4308 | 0.1954 | 0.0275 |
| Smoking Duration | 0.0091 | 0.0057 | 0.1103 |

**Dependent variable: Changed Interest in Sex**

| Covariates | Estimate | Standard error | p-value |
|---|---|---|---|
| AAE | -0.0487 | 0.0085 | $<1x10^{-5}$ |
| Gender | -0.6888 | 0.1536 | $<1x10^{-5}$ |
| Disease Duration | 0.0239 | 0.0148 | 0.1064 |
| Heart Diseases (binary) | 0.3482 | 0.1986 | 0.0795 |
| Smoking Duration | 0.0113 | 0.0059 | 0.0574 |

**Supplementary Table S10.** Motor symptoms associated with smoking in regression models including potential comorbidities.

| | | Heart Diseases | | | Lung Diseases | | |
|---|---|---|---|---|---|---|---|
| | | **Smoking** | | | | | |
| | | Yes/No | Dosage | Duration | Yes/No | Dosage | Duration |
| Tremor | n | 5249 | 4183 | 872 | 5245 | 4180 | 872 |
| | p-value | 0.1666 | 0.3558 | 0.4804 | 0.1477 | 0.3326 | 0.4715 |
| | β | 0.0967 | 0.0035 | -0.0047 | 0.1012 | 0.0037 | -0.0049 |
| Speech | n | 5252 | 4185 | 872 | 5248 | 4182 | 872 |
| | p-value | 0.1640 | **0.0076** | 0.0526 | 0.1672 | **0.0078** | 0.0591 |
| | β | 0.0829 | 0.0087 | 0.0115 | 0.0823 | 0.0087 | 0.0113 |
| Saliva and Drooling | n | 5252 | 4185 | 872 | 5248 | 4182 | 872 |
| | p-value | **0.0142** | **0.0028** | 0.0964 | **0.0151** | **0.0031** | 0.1609 |
| | β | 0.1427 | 0.0094 | 0.0094 | 0.1415 | 0.0093 | 0.0080 |
| Chewing and Swallowing | n | 5252 | 4185 | 872 | 5248 | 4182 | 872 |
| | p-value | **0.0004** | **$<1x10^{-5}$** | 0.0765 | **0.0004** | **$<1x10^{-5}$** | 0.1281 |
| | β | 0.2140 | 0.0169 | 0.0101 | 0.2162 | 0.0171 | 0.0088 |
| Walking and Balance | n | 5249 | 4183 | 872 | 5245 | 4180 | 872 |
| | p-value | 0.3366 | **0.0075** | **$9x10^{-5}$** | 0.3210 | **0.0064** | **0.0003** |
| | β | 0.0604 | 0.0094 | 0.0268 | 0.0625 | 0.0096 | 0.0241 |
| Freezing | n | 5249 | 4183 | 872 | 5245 | 4180 | 872 |
| | p-value | **0.0313** | **0.0065** | **$2x10^{-5}$** | **0.0274** | **0.0087** | **$9x10^{-5}$** |
| | β | 0.1395 | 0.0092 | 0.0270 | 0.1432 | 0.0088 | 0.0247 |
| Getting up | n | 5249 | 4183 | 872 | 5249 | 4180 | 872 |
| | p-value | 0.1130 | **0.0090** | **$<1x10^{-5}$** | 0.1085 | **0.0094** | **$<1x10^{-5}$** |
| | β | 0.1015 | 0.0093 | 0.0356 | 0.1029 | 0.0093 | 0.0349 |

P-value (exploratory): Multivariate regression to predict the respective motor symptoms adjusted for covariates by including AAE, gender, disease duration (time between AAO and current age), and comorbidities in the model.

**Supplementary Table S11.** Non-motor symptoms associated with smoking in regression models including potential comorbidities.

| | | Heart Diseases | | | Lung Diseases | | |
|---|---|---|---|---|---|---|---|
| | | **Smoking** | | | | | |
| | | Yes/No | Dosage | Duration | Yes/No | Dosage | Duration |
| Constipation | n | 5272 | 4200 | 876 | 5268 | 4197 | 876 |
| | p-value | 0.2324 | 0.2212 | 0.6463 | 0.2215 | 0.2081 | 0.6950 |
| | β | 0.0686 | 0.0037 | 0.0025 | 0.0702 | 0.0038 | 0.0022 |
| Unexplained Pains | n | 5271 | 4200 | 876 | 5267 | 4197 | 876 |
| | p-value | **$<1x10^{-5}$** | **0.0005** | **0.0205** | **$<1x10^{-5}$** | **0.0005** | **0.0224** |
| | β | 0.2695 | 0.0110 | 0.0132 | 0.2710 | 0.0109 | 0.0131 |
| Problems Remembering | n | 5271 | 4200 | 876 | 5267 | 4197 | 876 |
| | p-value | **0.0002** | **0.0002** | **0.0018** | **0.0001** | **$9x10^{-5}$** | **0.0047** |
| | β | 0.2165 | 0.0117 | 0.0176 | 0.2217 | 0.0120 | 0.0161 |
| Feeling Sad | n | 5269 | 4198 | 876 | 5265 | 4195 | 876 |
| | p-value | **$<1x10^{-5}$** | **$<1x10^{-5}$** | 0.0807 | **$<1x10^{-5}$** | **$<1x10^{-5}$** | 0.1103 |
| | β | 0.3200 | 0.0145 | 0.0099 | 0.3243 | 0.0149 | 0.0091 |
| Anxiety | n | 5269 | 4198 | 876 | 5265 | 4195 | 876 |
| | p-value | **$<1x10^{-5}$** | **$<1x10^{-5}$** | 0.4953 | **$<1x10^{-5}$** | **$<1x10^{-5}$** | 0.5394 |
| | β | 0.2969 | 0.0157 | 0.0039 | 0.2986 | 0.0158 | 0.0035 |
| Changed Interest in Sex | n | 5269 | 4198 | 876 | 5265 | 4195 | 876 |
| | p-value | **0.0013** | 0.2643 | 0.0574 | **0.0014** | 0.2353 | **0.0287** |
| | β | 0.1973 | 0.0036 | 0.0113 | 0.1951 | 0.0038 | 0.0131 |
| Light-headedness | n | 5268 | 4197 | 876 | 5264 | 4194 | 876 |
| | p-value | **0.0007** | **0.0003** | 0.1949 | **0.0005** | **0.0002** | 0.1439 |
| | β | 0.1953 | 0.0111 | 0.0072 | 0.2011 | 0.0115 | 0.0082 |

P-value (exploratory): Multivariate regression to predict the respective non-motor symptoms adjusted for covariates by including AAE, gender, disease duration (time between AAO and current age), and comorbidities in the model.

**Supplementary Table S12.** Symptoms related to mood associated with smoking status. Percentage of patients stratified by smoking status and symptoms related to mood.

| | | Smoking | | |
|---|---|---|---|---|
| | | yes | no | p-value |
| Depression | yes | 29.8% (n=627) | 24.2% (n=799) | **<1x10$^{-5}$** |
| | no | 70.2% (n=1475) | 75.8% (n=2509) | |
| Anxiety | yes | 31.5% (n=661) | 27.2% (n=900) | **2x10$^{-5}$** |
| | no | 68.5% (n=1440) | 72.8% (n=2409) | |
| Dropped many activities and interests | yes | 40.1% (n=837) | 33.0% (n=1087) | **<1x10$^{-5}$** |
| | no | 59.9% (n=1252) | 67.0% (n=2204) | |
| Life feels empty | yes | 16.0% (n=333) | 13.2% (n=433) | **0.0004** |
| | no | 84.0% (n=1751) | 86.8% (n=2858) | |
| Getting bored often | yes | 29.4% (n=615) | 22.3% (n=734) | **<1x10$^{-5}$** |
| | no | 70.6% (n=1475) | 77.7% (n=2558) | |
| Being afraid something bad could happen | yes | 25.0% (n=521) | 22.5% (n=739) | **0.0025** |
| | no | 75.0% (n=1559) | 77.5% (n=2551) | |
| Feeling helpless often | yes | 21.8% (n=453) | 17.8% (n=584) | **2x10$^{-5}$** |
| | no | 78.2% (n=1628) | 82.2% (n=2703) | |
| Prefer staying at home | yes | 54.6% (n=1136) | 50.0% (n=1644) | **0.0007** |
| | no | 45.4% (n=946) | 50.0% (n=1645) | |
| Feeling to have more memory problems than other people | yes | 28.4% (n=592) | 25.0% (n=823) | **0.0019** |
| | no | 71.6% (n=1494) | 75.0% (n=2472) | |
| Feeling pretty worthless | yes | 17.0% (n=354) | 14.1% (n=462) | **0.0002** |
| | no | 83.0% (n=1727) | 85.9% (n=2821) | |
| Feeling that situation is hopeless | yes | 14.8% (n=308) | 11.3% (n=372) | **<1x10$^{-5}$** |
| | no | 85.2% (n=1767) | 88.7% (n=2912) | |

P-value (exploratory): Multivariate regression to predict the respective mood symptoms adjusted for covariates by including AAE, gender, and disease duration (time between AAO and current age).

**Supplementary Table S13.** Generalized linear models on mood. Regression models for symptoms related to mood and associated with smoking in the Fox Insight cohort.

**Dependent variable: Depression**

| Covariates | Estimate | Standard error | p-value |
|---|---|---|---|
| AAE | -0.0176 | 0.0035 | $<1x10^{-5}$ |
| Gender | 0.3748 | 0.0638 | $<1x10^{-5}$ |
| Disease Duration | 0.0065 | 0.0060 | 0.2815 |
| Smoking (binary) | 0.3362 | 0.0649 | $<1x10^{-5}$ |

**Dependent variable: Anxiety**

| Covariates | Estimate | Standard error | p-value |
|---|---|---|---|
| AAE | -0.0284 | 0.0034 | $<1x10^{-5}$ |
| Gender | 0.3113 | 0.0621 | $<1x10^{-5}$ |
| Disease Duration | 0.0064 | 0.0059 | 0.2788 |
| Smoking (binary) | 0.2748 | 0.0636 | $2x10^{-5}$ |

**Dependent variable: Dropped many activities and interests**

| Covariates | Estimate | Standard error | p-value |
|---|---|---|---|
| AAE | 0.0034 | 0.0033 | 0.2995 |
| Gender | -0.0815 | 0.0585 | 0.1636 |
| Disease Duration | 0.0360 | 0.0057 | $<1x10^{-5}$ |
| Smoking (binary) | 0.3283 | 0.0598 | $<1x10^{-5}$ |

**Dependent variable: Feeling that life feels empty**

| Covariates | Estimate | Standard error | p-value |
|---|---|---|---|
| AAE | -0.0190 | 0.0043 | $1x10^{-5}$ |
| Gender | -0.0355 | 0.0798 | 0.6564 |
| Disease Duration | 0.0161 | 0.0074 | 0.0291 |
| Smoking (binary) | 0.2843 | 0.0809 | 0.0004 |

**Dependent variable: Getting bored often**

| Covariates | Estimate | Standard error | p-value |
|---|---|---|---|
| AAE | -0.0287 | 0.0036 | $<1x10^{-5}$ |
| Gender | -0.2916 | 0.0654 | $<1x10^{-5}$ |
| Disease Duration | 0.0334 | 0.0060 | $<1x10^{-5}$ |
| Smoking (binary) | 0.4263 | 0.0661 | $<1x10^{-5}$ |

**Dependent variable: Being afraid something bad could happen**

| Covariates | Estimate | Standard error | p-value |
|---|---|---|---|
| AAE | -0.0284 | 0.0036 | $<1x10^{-5}$ |
| Gender | 0.2330 | 0.0661 | 0.0004 |
| Disease Duration | -0.0188 | 0.0070 | 0.0069 |
| Smoking (binary) | 0.2050 | 0.0677 | 0.0025 |

**Dependent variable: Feeling helpless often**

| Covariates | Estimate | Standard error | p-value |
|---|---|---|---|
| AAE | -0.0109 | 0.0039 | 0.0050 |
| Gender | 0.0886 | 0.0708 | 0.2108 |
| Disease Duration | 0.0329 | 0.0064 | $<1x10^{-5}$ |
| Smoking (binary) | 0.3099 | 0.0720 | $2x10^{-5}$ |

**Dependent variable: Prefer staying at home**

| Covariates | Estimate | Standard error | p-value |
|---|---|---|---|
| AAE | -0.0105 | 0.0031 | 0.0007 |
| Gender | -0.1151 | 0.0559 | 0.0394 |
| Disease Duration | 0.0051 | 0.0055 | 0.3462 |
| Smoking (binary) | 0.1955 | 0.0576 | 0.0007 |

**Dependent variable: Feeling to have more memory problems than other people**

| Covariates | Estimate | Standard error | p-value |
|---|---|---|---|
| AAE | -0.0235 | 0.0035 | $<1x10^{-5}$ |
| Gender | -0.4046 | 0.0643 | $<1x10^{-5}$ |
| Disease Duration | 0.0102 | 0.0061 | 0.0938 |
| Smoking (binary) | 0.2027 | 0.0652 | 0.0019 |

**Dependent variable: Feeling pretty worthless**

| Covariates | Estimate | Standard error | p-value |
|---|---|---|---|
| AAE | -0.0192 | 0.0042 | $<1x10^{-5}$ |
| Gender | 0.0245 | 0.0779 | 0.7536 |
| Disease Duration | 0.0279 | 0.0070 | $7x10^{-5}$ |
| Smoking (binary) | 0.2958 | 0.0791 | 0.0002 |

**Dependent variable: Feeling that situation is hopeless**

| Covariates | Estimate | Standard error | p-value |
|---|---|---|---|
| AAE | -0.0184 | 0.0046 | $6x10^{-5}$ |
| Gender | 0.1564 | 0.0838 | 0.0620 |
| Disease Duration | 0.0397 | 0.0072 | $<1x10^{-5}$ |
| Smoking (binary) | 0.3846 | 0.0848 | $<1x10^{-5}$ |

**Dependent variable: Depression**

| Covariates | Estimate | Standard error | p-value |
|---|---|---|---|
| AAE | -0.0153 | 0.0040 | 0.0001 |
| Gender | 0.4080 | 0.0727 | $<1x10^{-5}$ |
| Disease Duration | 0.0038 | 0.0070 | 0.5885 |
| Smoking Dosage | 0.0199 | 0.0033 | $<1x10^{-5}$ |

**Dependent variable: Anxiety**

| Covariates | Estimate | Standard error | p-value |
|---|---|---|---|
| AAE | -0.0269 | 0.0039 | $<1x10^{-5}$ |
| Gender | 0.3355 | 0.0703 | $<1x10^{-5}$ |
| Disease Duration | 0.0054 | 0.0068 | 0.4266 |
| Smoking Dosage | 0.0182 | 0.0032 | $<1x10^{-5}$ |

**Dependent variable: Dropped many activities and interests**

| Covariates | Estimate | Standard error | p-value |
|---|---|---|---|
| AAE | 0.0005 | 0.0037 | 0.8925 |
| Gender | -0.0813 | 0.0664 | 0.2206 |
| Disease Duration | 0.0395 | 0.0065 | $<1x10^{-5}$ |
| Smoking Dosage | 0.0165 | 0.0031 | $<1x10^{-5}$ |

**Dependent variable: Feeling that life feels empty**

| Covariates | Estimate | Standard error | p-value |
|---|---|---|---|
| AAE | -0.0190 | 0.0049 | 0.0001 |
| Gender | 0.0257 | 0.0906 | 0.7764 |
| Disease Duration | 0.0144 | 0.0085 | 0.0916 |
| Smoking Dosage | 0.0181 | 0.0038 | $<1x10^{-5}$ |

**Dependent variable: Getting bored often**

| Covariates | Estimate | Standard error | p-value |
|---|---|---|---|
| AAE | -0.0313 | 0.0041 | $<1x10^{-5}$ |
| Gender | -0.2616 | 0.0744 | 0.0004 |
| Disease Duration | 0.0330 | 0.0069 | $<1x10^{-5}$ |
| Smoking Dosage | 0.0187 | 0.0033 | $<1x10^{-5}$ |

**Dependent variable: Being afraid something bad could happen**

| Covariates | Estimate | Standard error | p-value |
|---|---|---|---|
| AAE | -0.0267 | 0.0040 | $<1x10^{-5}$ |
| Gender | 0.3142 | 0.0744 | $2x10^{-5}$ |
| Disease Duration | -0.0203 | 0.0079 | 0.0101 |
| Smoking Dosage | 0.0126 | 0.0034 | 0.0002 |

**Dependent variable: Feeling helpless often**

| Covariates | Estimate | Standard error | p-value |
|---|---|---|---|
| AAE | -0.0116 | 0.0045 | 0.0096 |
| Gender | 0.1538 | 0.0812 | 0.0583 |
| Disease Duration | 0.0357 | 0.0074 | $<1x10^{-5}$ |
| Smoking Dosage | 0.0151 | 0.0036 | $3x10^{-5}$ |

**Dependent variable: Prefer staying at home**

| Covariates | Estimate | Standard error | p-value |
|---|---|---|---|
| AAE | -0.0122 | 0.0035 | 0.0004 |
| Gender | -0.0469 | 0.0628 | 0.4552 |
| Disease Duration | 0.0066 | 0.0062 | 0.2882 |
| Smoking Dosage | 0.0150 | 0.0032 | $<1x10^{-5}$ |

**Dependent variable: Feeling to have more memory problems than other people**

| Covariates | Estimate | Standard error | p-value |
|---|---|---|---|
| AAE | -0.0250 | 0.0039 | $<1x10^{-5}$ |
| Gender | -0.3760 | 0.0725 | $<1x10^{-5}$ |
| Disease Duration | 0.0122 | 0.0069 | 0.0779 |
| Smoking Dosage | 0.0105 | 0.0033 | 0.0014 |

**Dependent variable: Feeling pretty worthless**

| Covariates | Estimate | Standard error | p-value |
|---|---|---|---|
| AAE | -0.0210 | 0.0049 | $2x10^{-5}$ |
| Gender | 0.0356 | 0.0890 | 0.6894 |
| Disease Duration | 0.0337 | 0.0080 | $3x10^{-5}$ |
| Smoking Dosage | 0.0185 | 0.0038 | $<1x10^{-5}$ |

**Dependent variable: Feeling that situation is hopeless**

| Covariates | Estimate | Standard error | p-value |
|---|---|---|---|
| AAE | -0.0203 | 0.0053 | 0.0001 |
| Gender | 0.1846 | 0.0966 | 0.0562 |
| Disease Duration | 0.0429 | 0.0082 | $<1x10^{-5}$ |
| Smoking Dosage | 0.0172 | 0.0041 | $3x10^{-5}$ |

**Dependent variable: Depression**

| Covariates | Estimate | Standard error | p-value |
|---|---|---|---|
| AAE | -0.0262 | 0.0083 | 0.0017 |
| Gender | 0.1939 | 0.1528 | 0.2045 |
| Disease Duration | 0.0056 | 0.0156 | 0.7187 |
| Smoking Duration | 0.0123 | 0.0061 | 0.0422 |

**Dependent variable: Dropped many activities and interests**

| Covariates | Estimate | Standard error | p-value |
|---|---|---|---|
| AAE | -0.0109 | 0.0079 | 0.1678 |
| Gender | -0.1527 | 0.1435 | 0.2875 |
| Disease Duration | 0.0345 | 0.0146 | 0.0186 |
| Smoking Duration | 0.0253 | 0.0057 | $<1x10^{-5}$ |

**Dependent variable: Feeling that life feels empty**

| Covariates | Estimate | Standard error | p-value |
|---|---|---|---|
| AAE | -0.0262 | 0.0104 | 0.0118 |
| Gender | -0.1131 | 0.1953 | 0.5626 |
| Disease Duration | 0.0215 | 0.0184 | 0.2429 |
| Smoking Duration | 0.0230 | 0.0075 | 0.0021 |

**Dependent variable: Getting bored often**

| Covariates | Estimate | Standard error | p-value |
|---|---|---|---|
| AAE | -0.0371 | 0.0087 | $2x10^{-5}$ |
| Gender | -0.5031 | 0.1619 | 0.0019 |
| Disease Duration | 0.0324 | 0.0153 | 0.0343 |
| Smoking Duration | 0.0412 | 0.0063 | $<1x10^{-5}$ |

**Dependent variable: Being afraid something bad could happen**

| Covariates | Estimate | Standard error | p-value |
|---|---|---|---|
| AAE | -0.0395 | 0.0087 | $<1x10^{-5}$ |
| Gender | 0.0247 | 0.1619 | 0.8786 |
| Disease Duration | -0.0443 | 0.0195 | 0.0231 |
| Smoking Duration | 0.0184 | 0.0064 | 0.0039 |

**Dependent variable: Feeling helpless often**

| Covariates | Estimate | Standard error | p-value |
|---|---|---|---|
| AAE | -0.0278 | 0.0095 | 0.0036 |
| Gender | -0.3514 | 0.1790 | 0.0496 |
| Disease Duration | 0.0366 | 0.0177 | 0.0390 |
| Smoking Duration | 0.0296 | 0.0068 | $1x10^{-5}$ |

**Dependent variable: Prefer staying at home**

| Covariates | Estimate | Standard error | p-value |
|---|---|---|---|
| AAE | -0.0246 | 0.0079 | 0.0019 |
| Gender | -0.2025 | 0.1406 | 0.1499 |
| Disease Duration | 0.0054 | 0.0144 | 0.7049 |
| Smoking | 0.0179 | 0.0057 | 0.0017 |

**Dependent variable: Feeling to have more memory problems than other people**

| Covariates | Estimate | Standard error | p-value |
|---|---|---|---|
| AAE | -0.0259 | 0.0085 | 0.0022 |
| Gender | -0.2679 | 0.1582 | 0.0903 |
| Disease Duration | -0.0203 | 0.0173 | 0.2403 |
| Smoking Duration | 0.0178 | 0.0062 | 0.0038 |

**Dependent variable: Feeling pretty worthless**

| Covariates | Estimate | Standard error | p-value |
| --- | --- | --- | --- |
| AAE | -0.0351 | 0.0102 | 0.0006 |
| Gender | -0.2937 | 0.1918 | 0.1256 |
| Disease Duration | 0.0342 | 0.0175 | 0.0506 |
| Smoking Duration | 0.0370 | 0.0072 | $<1x10^{-5}$ |

**Dependent variable: Feeling hopeless**

| Covariates | Estimate | Standard error | p-value |
| --- | --- | --- | --- |
| AAE | -0.0367 | 0.0108 | 0.0007 |
| Gender | 0.1368 | 0.2033 | 0.5011 |
| Disease Duration | 0.0241 | 0.0195 | 0.2158 |
| Smoking Duration | 0.0304 | 0.0078 | 0.0001 |

**Supplementary Table S14.** Generalized linear models on symptoms related to mood including potential comorbidities. Regression models for symptoms related to mood with a change in outcome for smoking in the Fox Insight cohort while adjusting for potential comorbidities.

| | | **Heart Diseases** | | | **Lung Diseases** | | |
|---|---|---|---|---|---|---|---|
| | | **Smoking** | | | | | |
| | | Yes/No | Dosage | Duration | Yes/No | Dosage | Duration |
| Depression | n | 5213 | 4143 | 859 | 5213 | 4143 | 859 |
| | p-value | **<1x10$^{-5}$** | **<1x10$^{-5}$** | 0.0544 | **<1x10$^{-5}$** | **<1x10$^{-5}$** | 0.0584 |
| | β | 0.3354 | 0.0197 | 0.0117 | 0.3405 | 0.0197 | 0.0116 |
| Anxiety | n | 5213 | 4144 | 859 | 5213 | 4144 | 859 |
| | p-value | **2x10$^{-5}$** | **<1x10$^{-5}$** | 0.4005 | **2x10$^{-5}$** | **<1x10$^{-5}$** | 0.3173 |
| | β | 0.2725 | 0.0179 | 0.0051 | 0.2713 | 0.0178 | 0.0061 |
| Dropped many activities and interests | n | 5189 | 4138 | 866 | 5188 | 4138 | 866 |
| | p-value | **<1x10$^{-5}$** | **<1x10$^{-5}$** | **1x10$^{-5}$** | **<1x10$^{-5}$** | **<1x10$^{-5}$** | **1x10$^{-5}$** |
| | β | 0.3234 | 0.0158 | 0.0249 | 0.3278 | 0.0161 | 0.0251 |
| Life feels empty | n | 5185 | 4137 | 864 | 5184 | 4137 | 864 |
| | p-value | **0.0006** | **1x10$^{-5}$** | **0.0034** | **0.0005** | **<1x10$^{-5}$** | **0.0018** |
| | β | 0.2773 | 0.0170 | 0.0220 | 0.2840 | 0.0180 | 0.0237 |
| Getting bored often | n | 5191 | 4137 | 866 | 5190 | 4139 | 866 |
| | p-value | **<1x10$^{-5}$** | **<1x10$^{-5}$** | **<1x10$^{-5}$** | **2x10$^{-5}$** | **<1x10$^{-5}$** | **<1x10$^{-5}$** |
| | β | 0.4187 | 0.0179 | 0.0403 | 0.4244 | 0.0187 | 0.0422 |
| Being afraid something bad could happen | n | 5179 | 4133 | 862 | 5179 | 4133 | 862 |
| | p-value | **0.0037** | **0.0007** | **0.0060** | **0.0026** | **0.0004** | **0.0076** |
| | β | 0.1972 | 0.0117 | 0.0176 | 0.2046 | 0.0122 | 0.0172 |
| Feeling helpless often | n | 5177 | 4130 | 862 | 5177 | 4130 | 862 |
| | p-value | **2x10$^{-5}$** | **7x10$^{-5}$** | **3x10$^{-5}$** | **2x10$^{-5}$** | **4x10$^{-5}$** | **2x10$^{-5}$** |
| | β | 0.3053 | 0.0143 | 0.0284 | 0.3105 | 0.0147 | 0.0293 |
| Prefer staying at home | n | 5180 | 4131 | 862 | 5180 | 4131 | 862 |
| | p-value | **0.0008** | **<1x10$^{-5}$** | **0.0015** | **0.0006** | **<1x10$^{-5}$** | **0.0020** |
| | β | 0.1938 | 0.0148 | 0.0183 | 0.1970 | 0.0149 | 0.0178 |
| Feeling to have more memory problems than other people | n | 5190 | 4142 | 866 | 5190 | 4142 | 866 |
| | p-value | **0.0018** | **0.0027** | **0.0046** | **0.0016** | **0.0020** | **0.0097** |
| | β | 0.2040 | 0.0100 | 0.0175 | 0.2069 | 0.0103 | 0.0161 |
| Feeling pretty worthless | n | 5173 | 4124 | 860 | 5173 | 4124 | 860 |
| | p-value | **0.0003** | **<1x10$^{-5}$** | **<1x10$^{-5}$** | **0.0002** | **<1x10$^{-5}$** | **<1x10$^{-5}$** |
| | β | 0.2860 | 0.0172 | 0.0358 | 0.2965 | 0.0184 | 0.0375 |
| Feeling that situation is hopeless | n | 5168 | 4125 | 860 | 5168 | 4125 | 860 |
| | p-value | **1x10$^{-5}$** | **0.0001** | **0.0001** | **<1x10$^{-5}$** | **4x10$^{-5}$** | **0.0001** |
| | β | 0.3749 | 0.0161 | 0.0298 | 0.3847 | 0.0171 | 0.0301 |

P-value (exploratory): Multivariate regression to predict the respective symptoms related to mood adjusted for covariates by including AAE, gender, disease duration (time between AAO and current age), and comorbidities in the model.

**Supplementary Table S15.** Generalized linear models on mood. Regression models for symptoms related to mood and significant for smoking in the Fox Insight cohort.

**Dependent variable: Depression**

| Covariates | Estimate | Standard error | p-value |
|---|---|---|---|
| AAE | -0.0291 | 0.0085 | 0.0007 |
| Gender | 0.2161 | 0.1542 | 0.1610 |
| Disease Duration | 0.0063 | 0.0155 | 0.6852 |
| Heart Diseases (binary) | 0.3338 | 0.2038 | 0.1015 |
| Smoking Duration | 0.0117 | 0.0061 | 0.0544 |

**Dependent variable: Depression**

| Covariates | Estimate | Standard error | p-value |
|---|---|---|---|
| AAE | -0.0264 | 0.0083 | 0.0016 |
| Gender | 0.1906 | 0.1529 | 0.2127 |
| Disease Duration | 0.0063 | 0.0155 | 0.6851 |
| Lung Diseases (binary) | 0.1839 | 0.2025 | 0.3638 |
| Smoking Duration | 0.0116 | 0.0061 | 0.0584 |

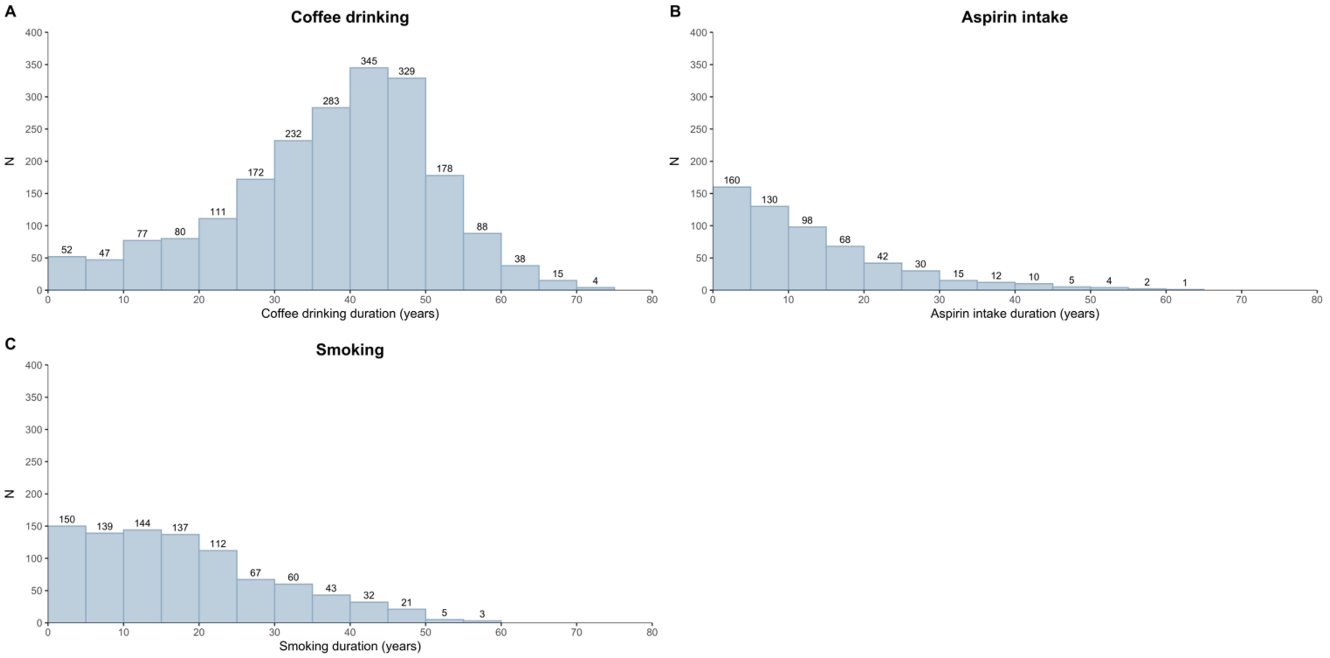


**Supplementary Figure S2.** Distribution of coffee drinking duration, aspirin intake duration, and smoking duration in the Fox Insight cohort. (**A**) Histogram of the coffee drinking duration in years. (**B**) Histogram of the aspirin intake duration in years. (**C**) Histogram of the smoking duration in years.

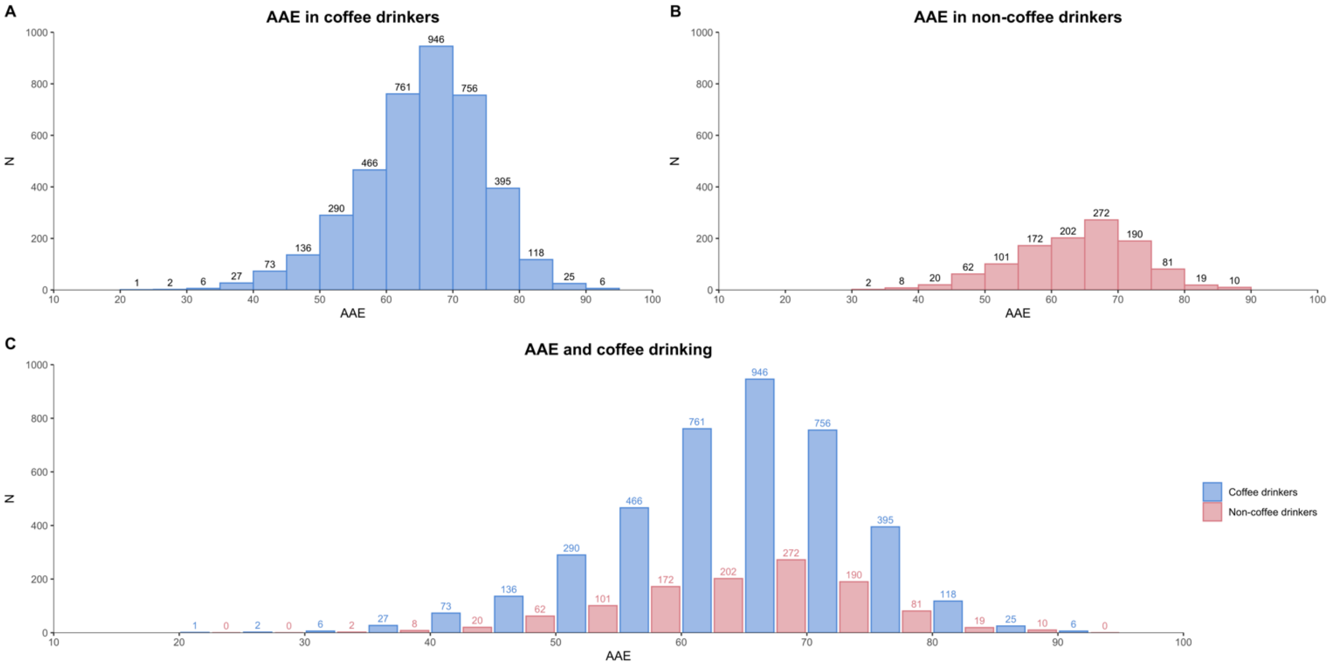


**Supplementary Figure S3.** Distribution of AAE in the subgroups of coffee drinkers and non-coffee drinkers. (**A**) Histogram of the AAE in the subgroup of coffee drinkers. (**B**) Histogram of the AAE in the subgroup of non-coffee drinkers (**C**) Histogram of the AAE in the subgroups of coffee drinkers and non-coffee drinkers.

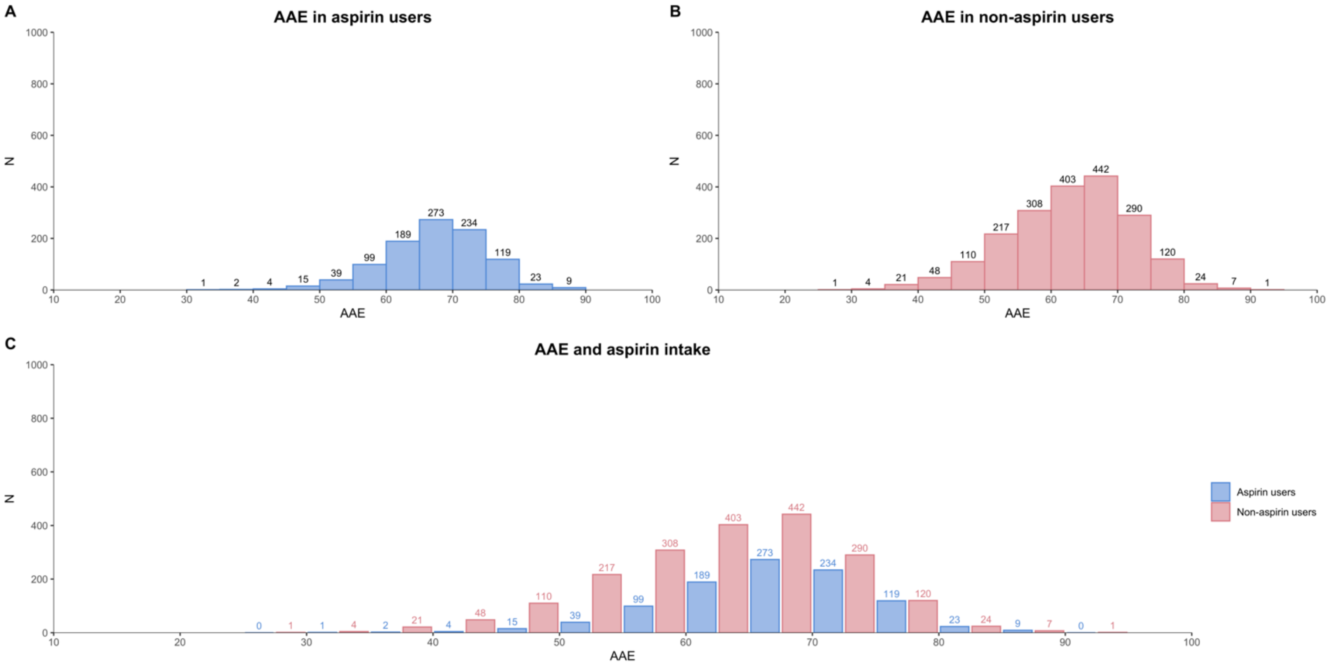


**Supplementary Figure S4.** Distribution of AAE in the subgroups of aspirin users and non-aspirin users. (**A**) Histogram of the AAE in the subgroup of aspirin users. (**B**) Histogram of the AAE in the subgroup of non-aspirin users (**C**) Histogram of the AAE in the subgroups of aspirin users and non-aspirin users.

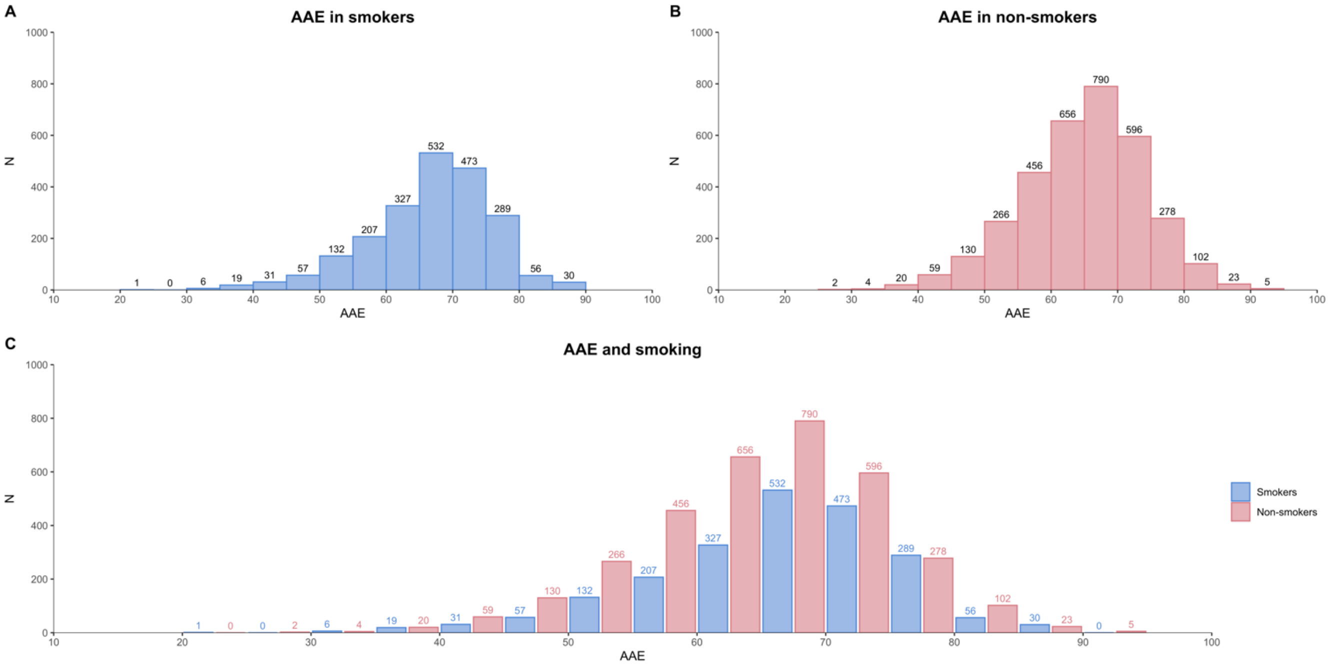


**Supplementary Figure S5.** Distribution of AAE in the subgroups of smokers and non-smokers. (**A**) Histogram of the AAE in the subgroup of smokers. (**B**) Histogram of the AAE in the subgroup of non-smokers (**C**) Histogram of the AAE in the subgroups of smokers and non-smokers.

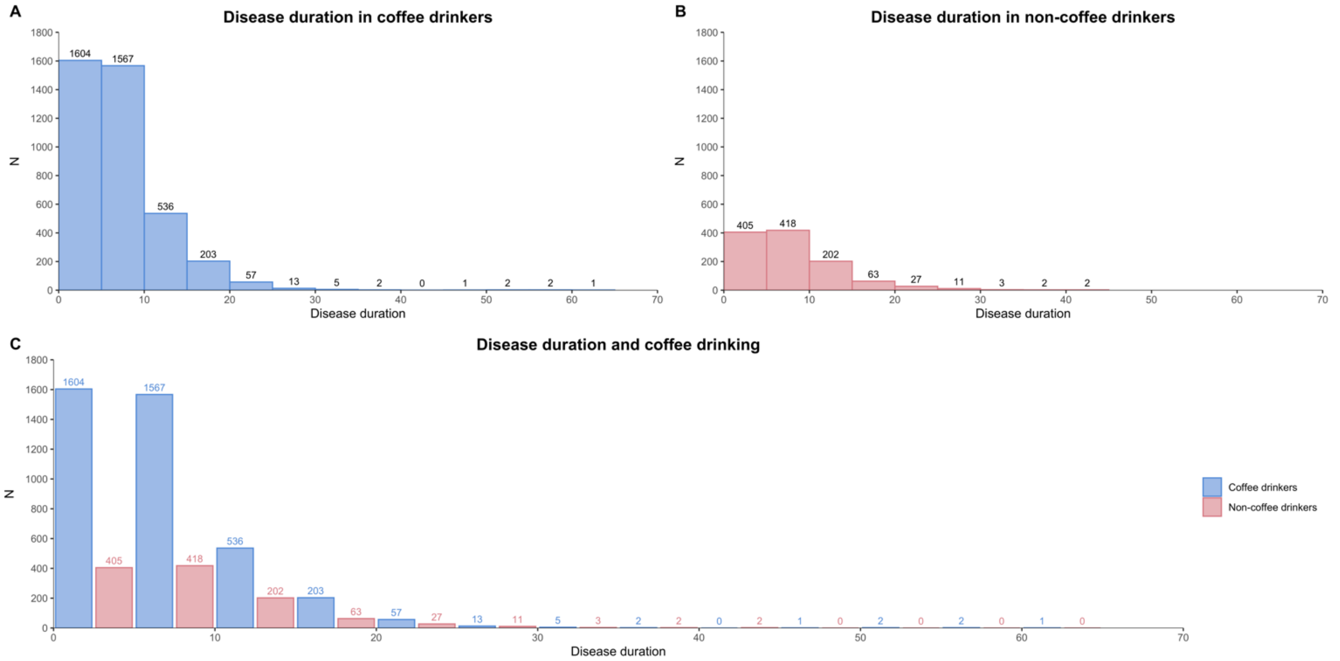


**Supplementary Figure S6.** Distribution of disease duration in the subgroups of coffee drinkers and non-coffee drinkers. (**A**) Histogram of the disease duration in the subgroup of coffee drinkers. (**B**) Histogram of disease duration in the subgroup of non-coffee drinkers (**C**) Histogram of disease duration in the subgroups of coffee drinkers and non-coffee drinkers.

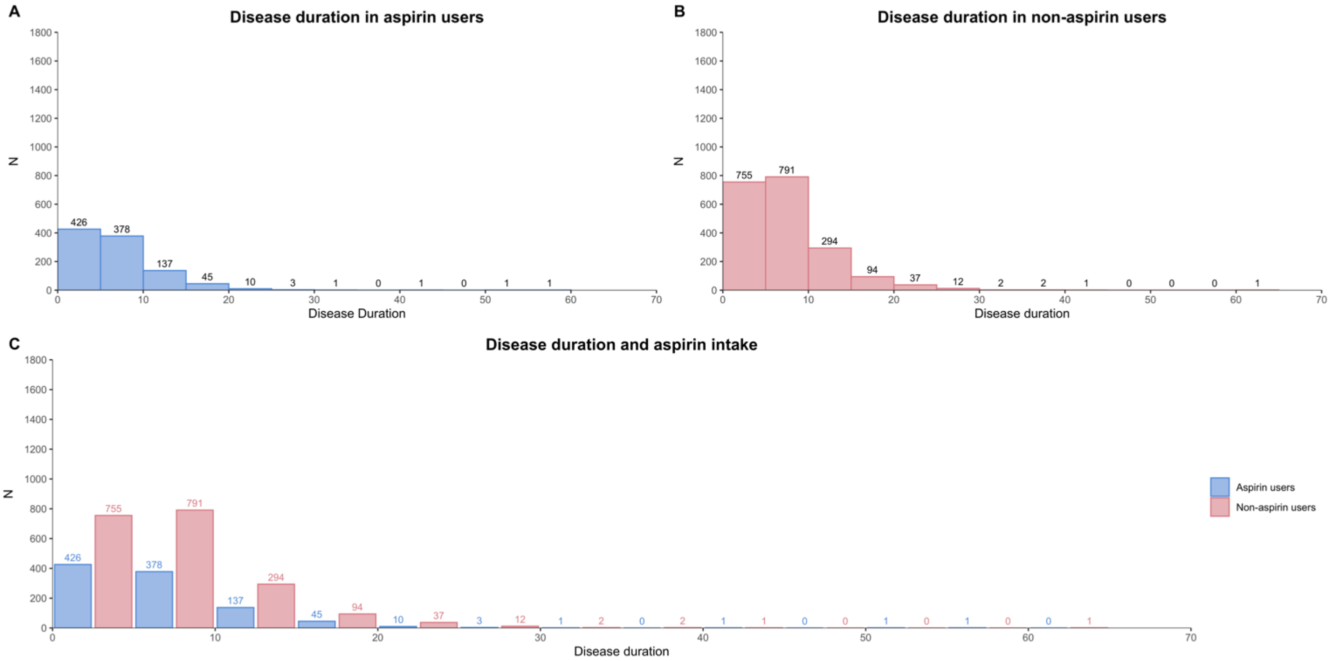


**Supplementary Figure S7.** Distribution of disease duration in the subgroups of aspirin users and non-aspirin users. (**A**) Histogram of the disease duration in the subgroup of aspirin users. (**B**) Histogram of the disease duration in the subgroup of non-aspirin users (**C**) Histogram of the disease duration in the subgroups of aspirin users and non-aspirin users.

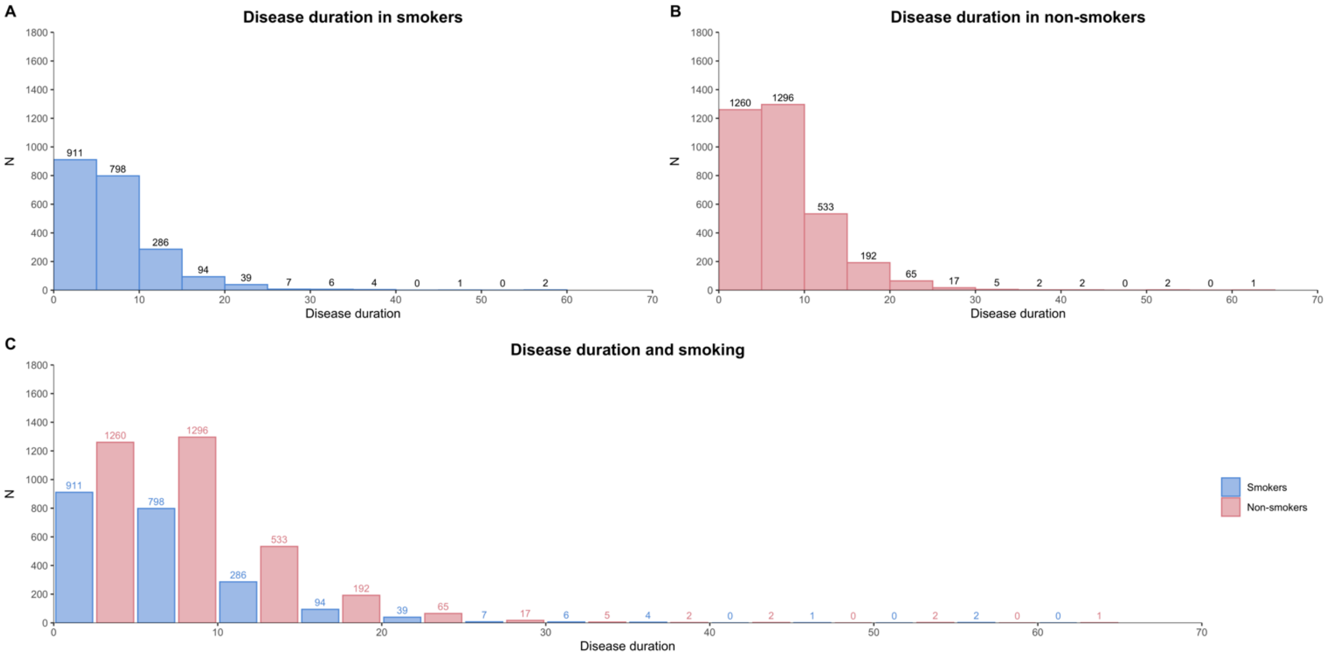


**Supplementary Figure S8.** Distribution of disease duration in the subgroups of smokers and non-smokers. (**A**) Histogram of the disease duration in the subgroup of smokers. (**B**) Histogram of the disease duration in the subgroup of non-smokers (**C**) Histogram of the disease duration in the subgroups of smokers and non-smokers.

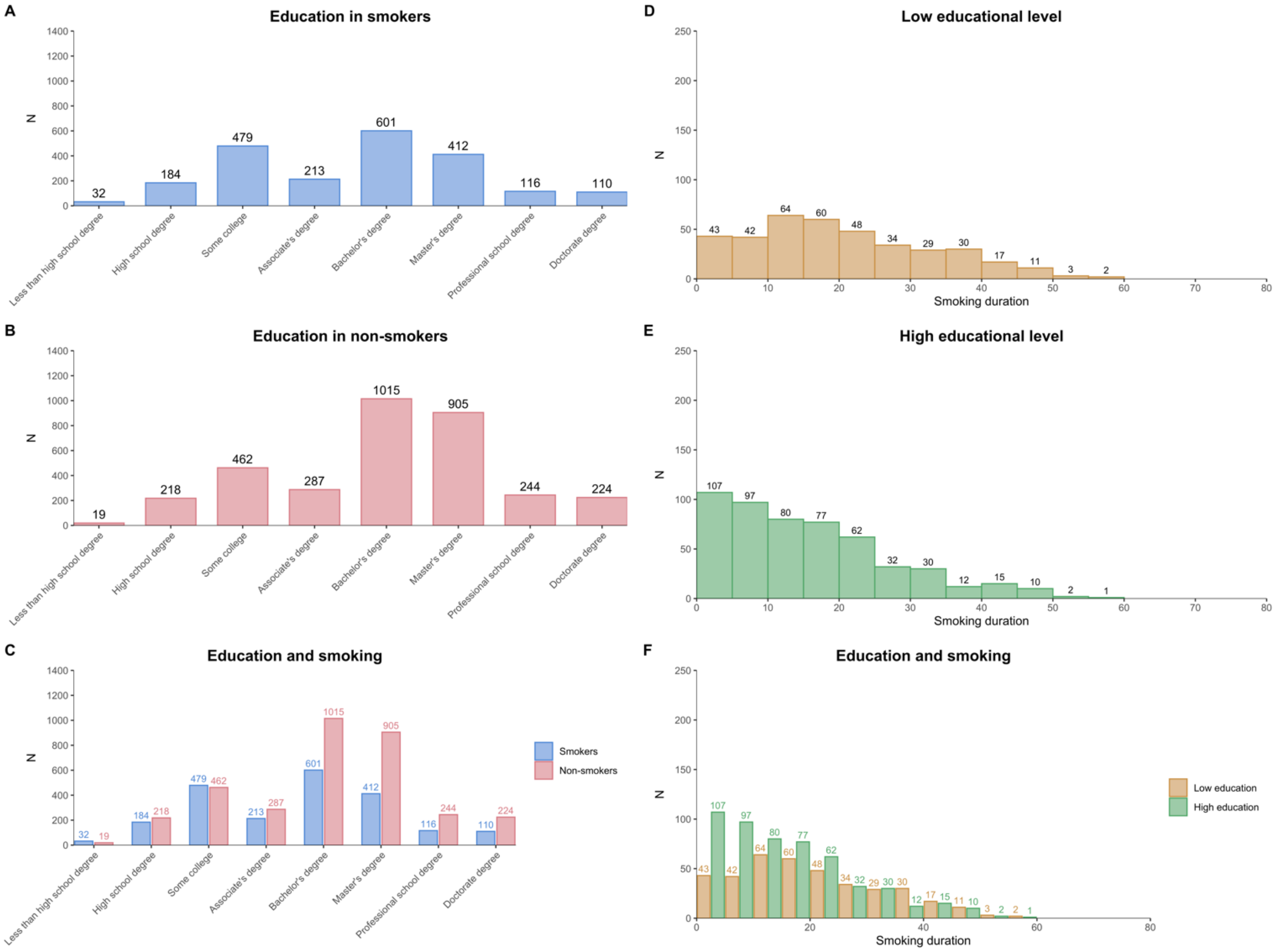


**Supplementary Figure S9.** Education and smoking. (**A**) Distribution of the educational level in the subgroup of smokers. (**B**) Distribution of the educational level in the subgroup of non-smokers (**C**) Distribution of the educational level in the subgroups of smokers and non-smokers. (**D**) Histogram of the smoking duration in the subgroup of smokers with a low educational level (i.e., less than a high school degree, high school degree, some college, and Associate's degree). (**E**) Histogram of the smoking duration in the subgroup of smokers with a high educational level (i.e., Bachelor's degree, Master's degree, professional school degree, and doctorate degree) (**F**) Histogram of the smoking duration in the subgroups of smokers with a low educational level and with a high educational level.

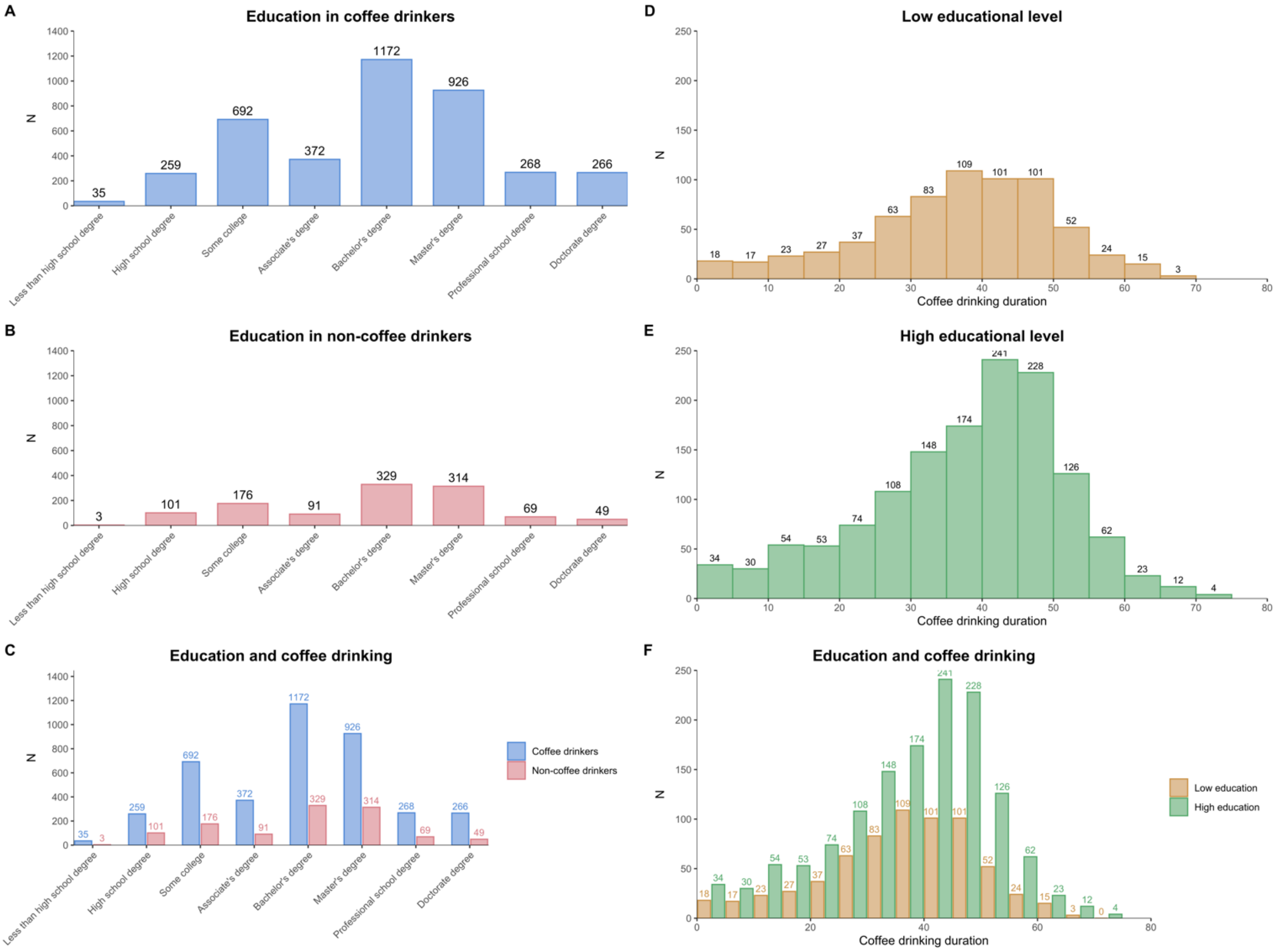


**Supplementary Figure S10.** Education and coffee drinking. (**A**) Distribution of the educational level in the subgroup of coffee drinkers. (**B**) Distribution of the educational level in the subgroup of non-coffee drinkers (**C**) Distribution of the educational level in the subgroups of coffee drinkers and non-coffee drinkers. (**D**) Histogram of the coffee drinking duration in the subgroup of coffee drinkers with a low educational level (i.e., less than a high school degree, high school degree, some college, and Associate's degree). (**E**) Histogram of the coffee drinking duration in the subgroup of coffee drinkers with a high educational level (i.e., Bachelor's degree, Master's degree, professional school degree, and doctorate degree) (**F**) Histogram of the coffee drinking duration in the subgroups of coffee drinkers with a low educational level and with a high educational level.

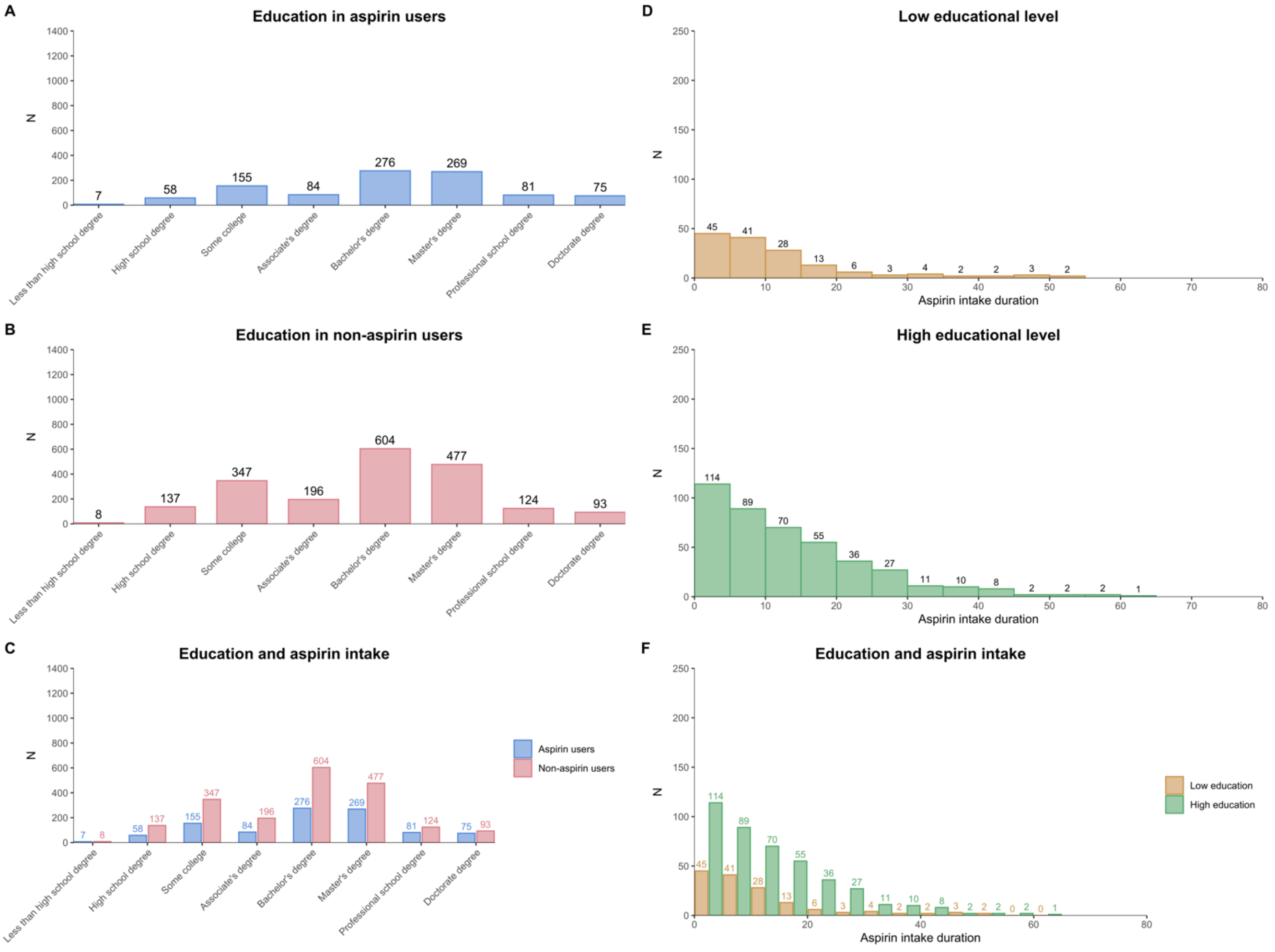


**Supplementary Figure S11.** Education and aspirin intake. (**A**) Distribution of the educational level in the subgroup of aspirin users. (**B**) Distribution of the educational level in the subgroup of non-aspirin users (**C**) Distribution of the educational level in the subgroups of aspirin users and non-aspirin users. (**D**) Histogram of the aspirin intake duration in the subgroup of aspirin users with a low educational level (i.e., less a than high school degree, high school degree, some college, and Associate's degree). (**E**) Histogram of the aspirin intake duration in the subgroup of aspirin users with a high educational level (i.e., Bachelor's degree, Master's degree, professional school degree, and doctorate degree) (**F**) Histogram of the aspirin intake duration in the subgroups of aspirin users with a low educational level and with a high educational level.

## References

1 Smolensky, L. *et al.* Fox Insight collects online, longitudinal patient-reported outcomes and genetic data on Parkinson's disease. *Sci Data* **7**, 67, doi:10.1038/s41597-020-0401-2 (2020).
